# Genetic Basis and Prognostic Value of Exercise QT Dynamics

**DOI:** 10.1161/CIRCGEN.119.002774

**Published:** 2020-06-11

**Authors:** Stefan van Duijvenboden, Julia Ramírez, William J. Young, Borbala Mifsud, Michele Orini, Andrew Tinker, Patricia B. Munroe, Pier D. Lambiase

**Affiliations:** 1 Institute of Cardiovascular Science, University College London, United Kingdom (S.v.D., J.R., M.O., P.D.L.).; 2 Clinical Pharmacology, William Harvey Research Institute (S.v.D., J.R., W.J.Y., B.M., M.O., A.T., P.B.M.), Barts & The London School of Medicine and Dentistry, Queen Mary University of London, United Kingdom.; 3 NIHR Barts Cardiovascular Biomedical Research Unit (A.T., P.B.M.), Barts & The London School of Medicine and Dentistry, Queen Mary University of London, United Kingdom.; 4 Barts Heart Centre, St Bartholomew’s Hospital, London, United Kingdom (W.J.Y., P.D.L.).; 5 College of Health and Life Sciences, Doha, Qatar (B.M.).

**Keywords:** cardiovascular disease, exercise, genome-wide association study, mortality, risk factors

## Abstract

**Background:**

Abnormal QT interval responses to heart rate (QT dynamics) is an independent risk predictor for cardiovascular disease in patients, but its genetic basis and prognostic value in a population-based cohort have not been investigated.

**Methods:**

QT dynamics during exercise and recovery were derived in 56 643 individuals from UK Biobank without a history of cardiovascular events. Genome-wide association studies were conducted to identify genetic variants and bioinformatics analyses were performed to prioritize candidate genes. The prognostic value of QT dynamics was evaluated for cardiovascular events (death or hospitalization) and all-cause mortality.

**Results:**

Heritability of QT dynamics during exercise and recovery were 10.7% and 5.4%, respectively. Genome-wide association studies identified 20 loci, of which 4 loci included genes implicated in mendelian long-QT syndrome. Five loci did not overlap with previously reported resting QT interval loci; candidate genes included *KCNQ4* and *KIAA1755*. Genetic risk scores were not associated with cardiovascular events in 357 882 unrelated individuals from UK Biobank. We also did not observe associations of QT dynamics during exercise and recovery with cardiovascular events. Increased QT dynamics during recovery was significantly associated with all-cause mortality in the univariate Cox regression analysis (hazard ratio, 1.09 [95% CI, 1.05–1.13], *P*=2.28×10^-5^), but the association was not significant after adjusting for clinical risk factors.

**Conclusions:**

QT interval dynamics during exercise and recovery are heritable markers but do not carry independent prognostic information for clinical outcomes in the UK Biobank, a population-based cohort. Their prognostic importance may relate to cardiovascular disease cohorts where structural heart disease or ischemia may influence repolarization dynamics. The strong overlap between QT dynamics and resting QT interval loci suggests common biological pathways; however, nonoverlapping loci suggests alternative mechanisms may exist that underlie QT interval dynamics.

## Abstract 2

Supplemental Digital Content is available in the text.

The ECG QT interval reflects the total duration of ventricular depolarization and repolarization and is a biomarker for cardiovascular risk and death, with an estimated heritability of ≈50% in twin-studies.^1^ Characteristically, the interval shortens and prolongs with increasing and decreasing heart rate respectively. Multiple lines of evidence indicate that abnormal dynamics of the QT interval, measured by the slope of the QT/RR profile, carries prognostic information in cardiac patients for cardiovascular mortality.^2–9^ The genetic architecture of QT dynamics has not been investigated and might inform biological mechanisms that underlie QT dynamics. Furthermore, the prognostic value of QT dynamics in a population-based cohort has not been evaluated.

An exercise stress test is a useful method to examine the dynamics of the QT interval in response to changes in heart rate. We first measured QT dynamics (approximated using RT dynamics) during exercise and recovery in 56 643 unselected individuals without a history of cardiovascular disease from the UKB study (UK Biobank). We next conducted genome-wide association studies (GWASs) in a subgroup of ≈52 000 individuals of European ancestry. We report for the first time the genetic architecture of QT dynamics, genetic risk score (GRS) results with cardiovascular outcomes, and evaluation of QT dynamics with cardiovascular events and all-cause mortality (ACM).

## Methods

**Figure 1.**
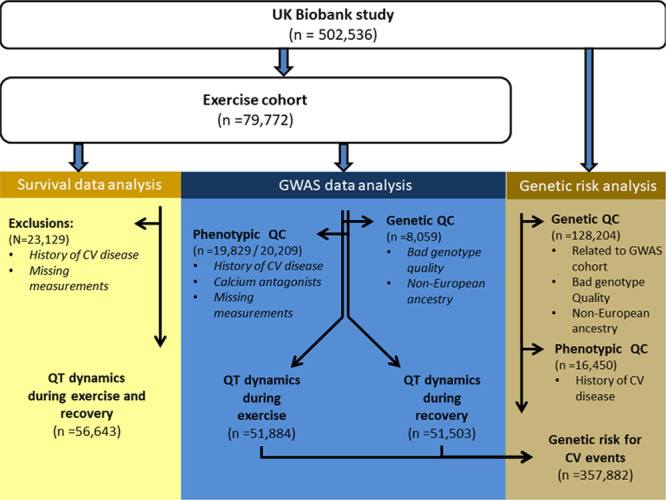
**Flowchart on selection of individuals for prognostic and genetic analyses of QT dynamics.** QT dynamics were derived from the exercise cohort. Yellow presents the data selection for the prognostic analysis, blue presents the selection for the genome-wide association studies (GWAS) and brown represents the individuals that were unrelated to individuals included in the GWAS to allow unbiased genetic risk analyses using the variants discovered in the GWAS. CV indicates cardiovascular; and QC, quality control.

The experimental design of the study is shown in Figure 1. Methods describing the derivation of QT dynamics, the genetic analysis, and survival analysis are available in the Data Supplement. The UKB study has approval from the North West Multi-Centre Research Ethics Committee, and all participants provided informed consent.^10^ Data used in this study were part of UKB application number 8256, and anonymized data and materials generated in this work have been returned to UKB and can be accessed per request.

## Results

### QT Dynamics During Exercise and Recovery Are Heritable Markers

**Figure 2.**
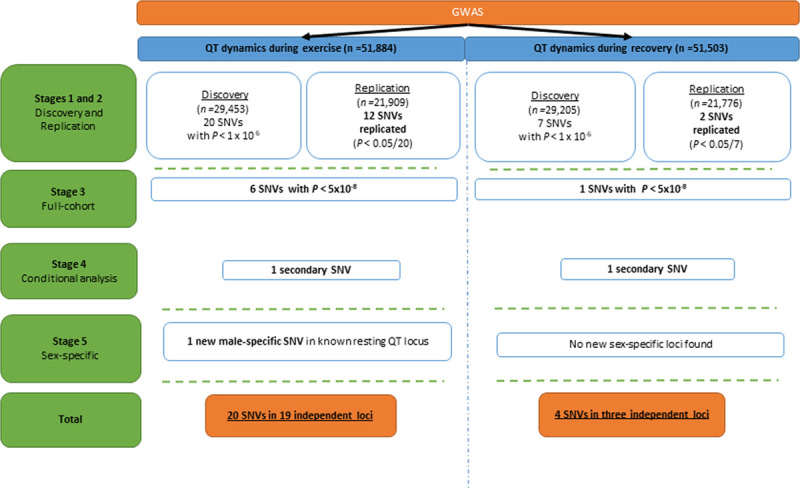
**Association results of the QT dynamics genome-wide association studies (GWAS) in the full data.** Note: To ensure there was no overlap between the discovery and the replication cohorts, we removed first and second-degree related individuals (kinship coefficient >0.88). SNV indicates a single-nucleotide variant.

An overview of the study results is provided in Figure 2. The demographics of the discovery and replication samples did not significantly differ (Table I in the Data Supplement). In the discovery phase, genome-wide association results of ≈9.8 million single-nucleotide variations (SNVs) from ≈30 000 individuals of European ancestry from UKB were analyzed for each trait, QT dynamics during exercise, and recovery. All SNVs with *P*<1×10^−6^ were compiled and these were organized into regions of 1 Mb. The SNV with the lowest *P* value in each 1 Mb region was selected as the lead SNV. In total, 20 lead SNVs for QT dynamics during exercise and 7 for QT dynamics during recovery were taken forward into replication in ≈22 000 unrelated individuals. Twelve SNVs for QT dynamics during exercise formally replicated (*P*≤0.05/20=0.0025) and all had concordant directions of effect (Table 1). For QT dynamics during recovery, 2 SNVs were formally replicated (*P*≤0.05/7=0.0071), all with concordant directions of effect (Table 2). We next performed a full data set GWAS for each trait (Methods). Six additional SNVs reached genome-wide significance for QT dynamics during exercise and 1 SNV for QT dynamics during recovery, all with concordant directions of effect in the full GWAS data set (Figure IV in the Data Supplement, Table 1). The QQ plots for both markers (Figure 5 in the Data Supplement) did not show evidence of population stratification or inflation. Regional plots are provided in Figure VI in the Data Supplement. Heritability estimations in the full dataset for QT dynamics during exercise and recovery were 10.7% and 5.4%, respectively, and their respective genetic correlation was 60%.

**Table 1.**
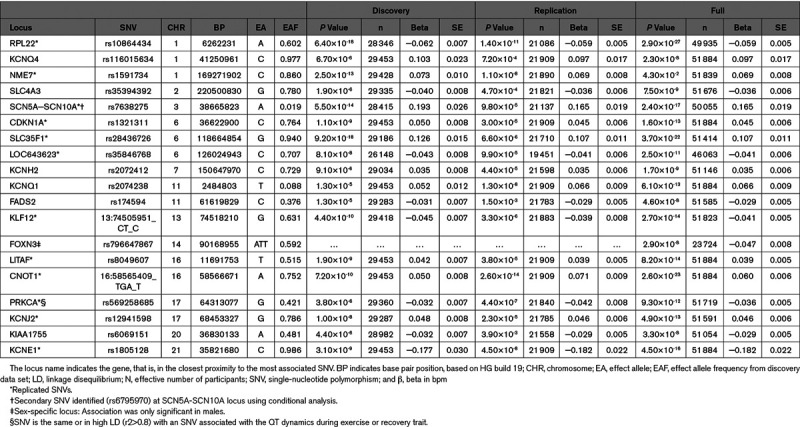
Loci Associated With QT Dynamics During Exercise

**Table 2.**
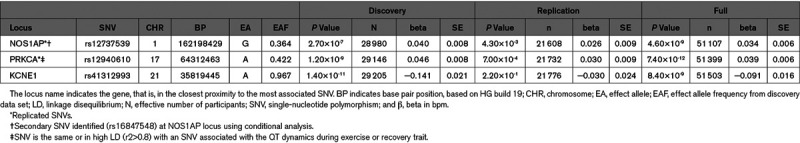
Loci Associated With QT Dynamics During Recovery

Conditional analysis revealed one secondary independent signal for QT dynamics during exercise at the *SCN5A-SCN10A* locus (rs6795970, Figure VIIA in the Data Supplement). This signal was ≈100 Kb downstream of the lead signal at this locus (rs7638275). For QT dynamics during recovery, one secondary signal was identified at the *NOS1AP* locus (rs16847548), at ≈163 kb upstream of the lead signal (rs12737539; Figure VIIB in the Data Supplement). Collectively, the lead and secondary variants explained 2.1% of the variance of QT dynamics during exercise (≈20% of heritability) and 0.6% (≈11% of heritability) for QT dynamics during recovery.

Sex-stratified analyses revealed one additional genome-wide significant locus (*FOXN3*) for QT dynamics during exercise (Table III and Figure VIB in the Data Supplement): this locus at chromosome 14 was only genome-wide significant (*P*≤5×10^-8^) in males (rs796647867, *P*=2.9×10^-8^). No sex-specific loci for QT dynamics during recovery were identified. Altogether, 19 novel loci were associated with QT dynamics during exercise and 3 with QT dynamics during recovery. Two loci (*PRKC*1 and *KCNE1*) were common to both; thus, we identified 20 novel unique loci for QT dynamics (Figure 2).

### Genetic Overlap Between QT Dynamics and Other ECG Markers

**Figure 3.**
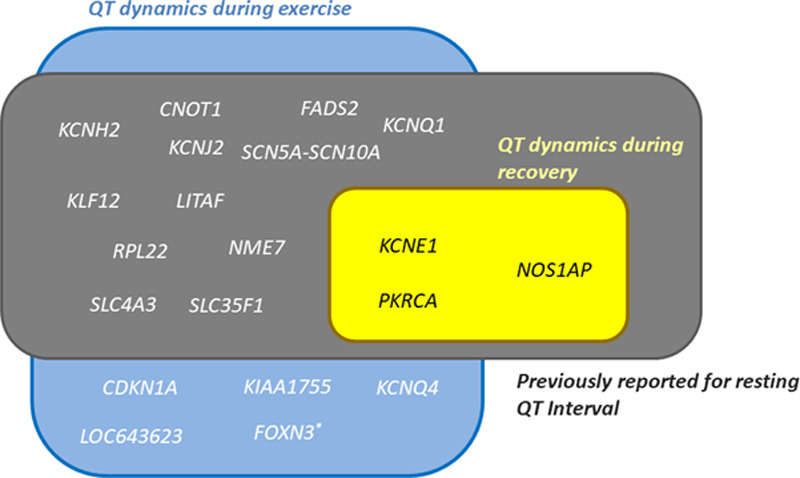
**Overlap between loci for QT dynamics during exercise (blue box) and recovery (yellow box) and reported loci for resting QT interval (black box).** A substantial proportion of the loci for QT dynamics overlapped loci previously reported for resting QT interval. Five loci (bold) for QT dynamics during exercise did not overlap with previously reported loci for resting QT interval. All loci for QT dynamics during recovery overlapped with resting QT interval loci. *Sex-specific (males) locus for QT dynamics during exercise.

Fifteen of the 20 loci discovered for QT dynamics overlapped with loci previously reported for resting QT interval from published GWAS (Figure 3 and Table IV in the Data Supplement). Two loci had reported genome-wide phenotype-genotype associations with another ECG marker: the *CDKN1A* locus with QRS and JT duration and the *KIAA1755* locus with resting heart rate (Table V in the Data Supplement).

### Functional Annotation of QT Dynamics Loci and Candidate Genes

The lead variants or close proxies at the 5 unique loci for QT dynamics during exercise (*KCNQ4*, *CDKN1A*, *LOC643623*, *KIAA1755, FOXN3*) were all annotated as either intronic or intergenic (Table VI in the Data Supplement), and no variants were associated with changes in expression levels of nearby genes in the Genotype-Tissue Expression database.^11^ We explored the regulatory potential of the associated variants at these 5 loci using Hi-C datasets and observed significant chromatin interaction (RegulomeDB score=2b) at *KIAA1755* with several genes in heart ventricles, brain, and adrenal gland (Table VII in the Data Supplement).

At the remaining 15 loci for QT dynamics 5 lead variants or their close proxies mapping to *SLC4A3*, *SCN5A-SCN10A*, *KCNH2*, *FADS2*, and *KCNE1* were annotated as missense variants (Table VI in the Data Supplement). The variant rs751489327, a close proxy of the lead variant at *CNOT1*, was annotated as a frameshift variant (Table VI in the Data Supplement). The associated variants at *KCNH2* and *KCNE1* were predicted to be possibly damaging or deleterious by either sorting intolerant from tolerant or polymorphism phenotyping.^12^ Using COLOC,^13^ we observed evidence for colocalization (posterior probability >75%) between significant gene expression (expression quantitative trait locus) and QT dynamics GWAS signals at 2 loci: *LITAF* and *PRKCA* in left ventricular and atrial appendage tissue, respectively (Table VIII in the Data Supplement). Interestingly, variants at the *PRKCA* locus were discovered for both QT dynamics traits, and both demonstrated high degree of colocalization with the expression quantitative trait locus signal (Figure VIII in the Data Supplement). The top expression quantitative trait locus variant for this locus in the heart left ventricle was rs11658550; this variant was in high linkage disequilibrium (*r*^2^>0.9) with the lead variants for QT dynamics during exercise (rs569258685) and recovery (rs12960410).

Data-driven expression prioritized integration for complex traits analysis indicated significant enrichment in heart and ventricular tissue (Table IXA in the Data Supplement). Several genes including *SCN5A*-*SCN10A*, *SLC35F1*, and *KCNQ1* were indicated as prioritized genes (Table IXB in the Data Supplement). Table X in the Data Supplement presents an overview of potential candidate genes at each locus from all bioinformatic analyses and literature review. For these candidate genes, we found significant enrichments across several gene ontology terms including voltage-gated channel activity and cardiac muscle cell action potential. Ventricular fibrillation and torsade de pointes were indicated as the human phenotypes. With fewer genes for QT dynamics during recovery, the enrichments included several ventricular membrane repolarization processes (Table XI in the Data Supplement).

### GRS Analyses

Given the limited number of variants associated with QT dynamics during recovery, we only tested the GRS for QT dynamics during exercise for cardiovascular outcome. The GRS was constructed using 18 lead and 1 secondary SNVs (Table 1) discovered in the full GWAS analysis. From the 357 822 included individuals, 18 732 (5.2%) had a cardiovascular event. The cardiovascular risk in the top 5% was not higher compared with the bottom 5% of the GRS distribution: odds ratio, 1.05 [95% CI, 0.96–1.16], *P*=0.3.

### Survival Analyses

We also assessed whether QT dynamics are associated with cardiovascular outcome and ACM. Among the 55 643 individuals without a previous history of cardiovascular events in the exercise UKB cohort (Figure 1), 1786 (3.2%) had a cardiovascular event, and 999 (1.8%) died (Table 3). QT dynamics during exercise and recovery were not significantly different for the cardiovascular event group but were significantly higher in the ACM group (median [interquartile range] values of 0.18 [0.07] versus 0.17 [0.07], *P*=0.04 for QT dynamics during exercise, and 0.12 [0.10] versus 0.11 [0.09], *P*=0.03, for QT dynamics during recovery). Only QT dynamics during recovery was a significant predictor of ACM in the univariate Cox regression analysis: hazard ratio, 1.09 (95% CI, 1.05–1.13), *P*=2.28×10^-5^, but this did not remain significant after adjusting for covariates (Table 4).

**Table 3.**
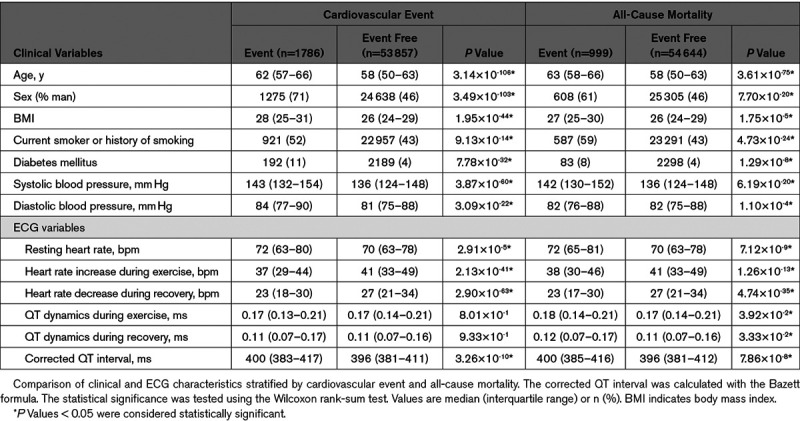
Baseline Characteristics Stratified by Outcome

**Table 4.**
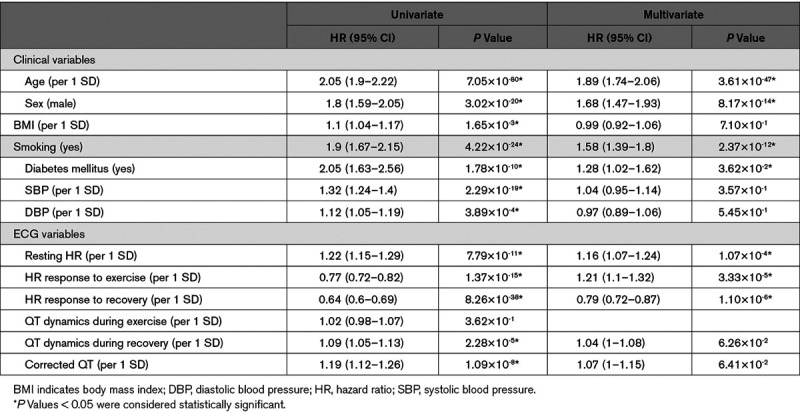
Univariate and Multivariate Association of QT Dynamics and Other Risk Markers With All-Cause Mortality

## Discussion

This is the first study to systematically investigate the genetic basis and prognostic value of QT dynamics during exercise and recovery in a large cohort of unselected individuals without a history of cardiovascular events. Our main findings are (1) QT dynamics during exercise has a significant heritable component (10%), with the recovery marker being less heritable (≈6%); (2) there is substantial genetic overlap between both QT dynamics markers and resting QT interval, and support for 5 loci for QT dynamics during exercise not previously reported for resting QT interval, and (3) QT dynamics during exercise and recovery were not associated with the occurrence of cardiovascular events or ACM in a population-based cohort.

### Genetic Architecture of Exercise QT Dynamics

We demonstrate for the first time that QT dynamics during exercise and recovery are heritable; however, compared with the resting QT interval, the heritability is less and environmental factors likely have a more significant. Substantial overlap between loci for QT dynamics and resting QT interval suggests important genetic overlap. We observed variants at loci containing genes previously established to cause monogenic long-QT syndrome and encoding ion channels or channel-interacting proteins (*KCNQ1*, *KCNH2*, *SCN5A*-*SCN10A*, and *KCNE1*). This may suggest that common variants in these genes do not only play a role in modulating resting QT interval but also QT dynamics, possibly by affecting rate-dependent channel kinetics. For example, *KCNQ1* and *KCNE1* are well known to confer kinetic properties on IKs currents required for rate adaptation of the cardiac action potential during exercise when β-adrenergic stimulation enhances IKs currents.^14^

Five loci did not overlap with previously reported resting QT interval loci and thus may indicate biological mechanisms that specifically underlie QT dynamics. For example, one interesting candidate gene is *KCNQ4*, which encodes the potassium voltage-gated channel subfamily Q member 4. *KCNQ4* is expressed in neurons and blood vessels and may also be involved in cardiac mitochondrial calcium handling.^15^ The neuronal function is best established in the cochlear, where mutations are associated with human dominant hereditary deafness,^16^ but this gene is widely expressed in other nerves and may regulate transmitter release in autonomic ganglia and nerve terminals. A second potential candidate gene at a different locus is *KIAA1755*, which is only characterized at the transcriptional level.^11^ Variants at this locus have previously been associated with resting heart rate^17^ and heart rate variability.^18^ *KIAA1755* is highly expressed in brain and nerve tissues and may play a role in the autonomic control of the heart.

From the loci that overlapped with resting QT interval, we found evidence for colocalization at the *PRKCA* locus between expression quantitative trait locus signals and GWAS signals for QT dynamics during exercise and recovery. This gene is an important regulator of cardiac contractility and *Ca*^2+^ handling in myocytes. Mechanistically, modulation of PKC (protein kinase C)-alpha activity affects dephosphorylation of the SERCA-2 (sarcoplasmic reticulum Ca^2+^ ATPase-2) pump inhibitory protein PLB (phospholamban), and alters sarcoplasmic reticulum Ca^2+^ loading and the Ca^2+^ transient. The regulation of cardiac contraction is critically important during exercise and recovery and is tightly coupled with the electrical processes of the heart, which may be reflected in the QT dynamics possibly through recognized Ca^2+^ dependent mechanisms and stretch influencing QT interval.^19^

Previous work has shown that ventricular repolarization is strongly modulated by heart rate but also independently by autonomic nervous activity^20^ and QT dynamics is thought to offer a more integrative measure of autonomic balance under stress than resting QT interval alone.^21^ This is supported by multiple lines of evidence showing that abnormal QT dynamics is an independent predictor of cardiovascular mortality in cardiac patients.^2–9^

The QT dynamics and the response of autonomic nervous activity have shown to be different between females and males.^22^ The variant rs796647867 at the *FOXN3* locus was only associated with QT dynamics during exercise in men, suggesting that the genetic basis for QT dynamics during exercise may differ between sexes. *FOXN3* is a candidate gene at the locus and is a member of the forkhead/winged-helix transcription factor family. The mechanism by which it could modulate QT dynamics is unclear.

### Prognostic Value of QT Dynamics in the General Population

The independent association between QT dynamics and cardiovascular events has been established in multiple studies including patients with heart failure,^8^ cardiomyopathy,^9^ and postmyocardial infarction^2^ but not in a population-based sample as interrogated in our study. We found that QT dynamics was not an independent predictor of cardiovascular events and ACM. A review of the previous studies indicates only one used exercise stress data to measure QT dynamics during recovery.^4^ According to this study, QT dynamics was a predictor of mortality in n=2994 cardiovascular patients. Reported values for QT dynamics were higher compared with our findings (0.39 versus 0.17 and 0.11, respectively). Increased QT dynamics are observed in patients at risk for cardiac death and arrhythmic events,^23^ and the fact that our values were lower may explain why QT dynamics were not associated with cardiovascular outcome or mortality in our cohort. It is possible that we measured lower values for QT dynamics because our population was healthier. However, it should be mentioned that they exercised at submaximal level only. It is well known that maximal workloads are superior in predicting cardiovascular risk and ACM.^24^ In addition, in our data the duration of the recovery period was shorter. The time-lag in the response of the QT interval to a sudden change in heart rate can take up to 2 minutes.^25^ The recovery period in our work lasted 1 minute, and it is, therefore, possible that the QT interval was not fully adapted resulting in a reduced QT/RR slope during recovery. QT interval dynamics reported in this work are specific for exercise and recovery. Importantly, QT dynamics has also shown to carry prognostic value when derived from the QT/RR relationship of 24-hour ECG recordings using linear regression between all QT and RR values. We did not have access to this data, but reported values in patients (0.17–0.22 for heart failure patients^8^ and 0.20 for cardiomyopathy^9^) were similar to QT dynamics during exercise observed in our cohort (0.17).

### Limitations

Several limitations in our study should be noted. First, from the 22 SNVs discovered, 14 formally replicated. The remaining identified SNVs we are reporting are from a GWAS of all samples and require formal replication in an independent data set. We also note our sample is relatively small compared to many other GWAS and this will limit power for discovery of loci. Second, the power to evaluate the predictive value of QT dynamics was limited as the number of events was low. This coupled with the small number of genetic variants (N=19) which only explained 2.1% of the variance of QT dynamics during exercise (≈20% of heritability), also affects the power of the GRS.

In summary, we demonstrate for the first time that QT dynamics during exercise and recovery are heritable markers. Its genetic basis largely overlaps with resting QT interval; however, there may be additional biological mechanisms that specifically underlie QT dynamics having identified 5 novel loci specific to these traits. QT dynamics during exercise and recovery were not independent predictors of cardiovascular events or ACM as opposed to cardiovascular disease cohorts where structural heart disease or myocardial ischemia affect repolarization dynamics. Future studies will be required to evaluate the prognostic value of the GRS in cardiovascular cohorts.

## Sources of Funding

This research has been conducted using the UKB (UK Biobank) Resource (application 8256) and is supported by grant MR/N025083/1, by the National Institutes of Health Research (NIHR) Cardiovascular Biomedical Centre at Barts and The London, Queen Mary University of London (QMUL), by the People Programme of the European Union’s Seventh Framework Programme grant n° 608765 and Marie Sklodowska-Curie grant n° 786833, by the University College London Hospital Biomedicine NIHR, Barts Heart Centre Biomedical Research Centre. Dr Young is funded by the Medical Research Council (Grant code MR/R017468/1). This research utilized Queen Mary’s Apocrita High-performance cluster facility, supported by QMUL Research-IT. http://doi.org/10.5281/zenodo.438045.

## Disclosure

None.

## Supplementary Material


