# Supplementary material for: Genetic Basis and Prognostic Value of Exercise QT Dynamics
Source: Circ Genom Precis Med. 2020 Jun 11;13(4):e002774. doi: 10.1161/CIRCGEN.119.002774 (PMC7439940; doi:10.1161/CIRCGEN.119.002774)
Supplement: Supplementary file 1 [file hcg-13-e002774-s001.pdf]

Supplemental Material

Supplemental Methods

Data selection of individuals

Genome-wide association studies (GWAS) and prognostic analyses were performed using data from the UK Biobank (UKB) study. UKB is a prospective study of ~500,000 volunteers, comprising even numbers of men and women aged 40-69 years on recruitment, with extensive baseline and follow-up clinical, biochemical, genetic and outcome measures[1]. The UKB study has approval from the North West Multi-Centre Research Ethics Committee, and all participants provided informed consent[1]. From the full UKB cohort, 79,772 individuals participated in an exercise stress test using a stationary bicycle with raw electrocardiographic (ECG) recordings available to measure QT dynamics.

Derivation of QT dynamics

The UKB exercise stress test followed a standardized protocol including a 15 s resting period (pre-test), followed by a 6 min exercise with gradual increasing workload, and a 1 min recovery period without pedalling. The changes of the QT interval during exercise are predominantly influenced by changes in ventricular repolarization, as heart rate-related changes in ventricular depolarization are minimal[2]. We therefore measured RT dynamics (changes in the R-wave to Tend interval) as an approximation of the QT dynamics (changes in the QRS onset to Tend interval) and use the term QT dynamics thereafter for RT dynamics[3]. Compared to the QT interval, the RT interval relies on the R-wave peak instead of the QRS onset, which can be detected more reliably improving the robustness of the measurements (Supplemental Fig. 1).

ECG recordings were pre-processed to extract heartbeats from each recording automatically. Details have been published previously[4]. Supplemental Fig. 2 shows how the QT dynamics during exercise and recovery were derived, which included the following steps:

(1) Construction of three averaged ECG waveforms by aligning and averaging all heart beats within a 15s window at three stages along the exercise stress test: (A) resting (pre-exercise, rest), (B) peak-exercise (ex), and (C) 50s recovery from exercise (rec).

(2) From each averaged ECG waveform, we measured the RT interval as the interval between the R-peak and the end of the T-wave using the tangent method[5] (Supplemental Fig. 1). The corresponding RR intervals were calculated as the median of all RR intervals within the 15s window.

(3) The changes in RT and RR intervals during exercise (ΔRT_{ex} and ΔRR_{ex}) and recovery (ΔRT_{rec} and ΔRR_{rec}) were then computed as:

$$\Delta RT_{ex} = RT_{rest} - RT_{ex}$$

$$\Delta RT_{rec} = RT_{rec} - RT_{ex}$$

$$\Delta RR_{ex} = RR_{rest} - RR_{ex}$$

$$\Delta RR_{rec} = RR_{rec} - RR_{ex}$$

(4) Finally, the QT dynamics were computed by dividing the changes in RT by the corresponding changes in RR interval:

$$\text{QT dynamics during exercise} \sim \frac{\Delta RT_{ex}}{\Delta RR_{ex}}$$

$$\text{QT dynamics during recovery} \sim \frac{\Delta RT_{rec}}{\Delta RR_{rec}}$$

Genetic quality control

To study the genetic basis and risk of QT dynamics, we used genotyped data of individuals from European ancestry from UKB. Genotyping was performed by UKB using the Applied Biosystems UK BiLEVE Axiom Array or the UKB AxiomTM Array[6]. SNVs were imputed centrally by UKB using the Haplotype Reference Consortium (HRC) and UK10K/1000Genomes haplotype resource panels[6]. We applied genetic quality control (QC) to exclude individuals with bad genotype quality, provided by UKB, i.e. high missingness or heterozygosity and discordance between the self-reported sex, and the sex inferred from the genotypes were excluded (N = 2,655)[6]. We then restricted our dataset to individuals of

European ancestry only (N = 71,713). This was achieved using the k-means function in R as a clustering algorithm to select clusters according to information from the first two principal components (PC1 and PC2). The k-means algorithm partitions the points into k groups such that the sum of squares from points to the assigned cluster centres is minimised. Then, we applied k-means separately to cluster according to each of PC1 and PC2, and initially only with k=4, for a 4-way clustering, to correspond to the 4 main ethnic clusters within UKB: White, African, Asian and Chinese. We then created an overall clustering, according to the intersections of the PC1-4means-clustering and the PC2-4means-clustering, so that participants were only categorised as "White" overall, if they were contained in the "White" cluster for both PC1 and PC2. Next, we created an overall "Mixed / Other" cluster, for any participants, whose clustering differed between PC1 and PC2. Finally, we combined the PCA ancestry clusters with the self-reported ethnicity. Individuals were only included if the results PCA-clustering results matched the self-reported ancestry.

Phenotypic quality control

We next excluded all individuals with pre-existing medical conditions known to affect QT dynamics. This included atrial fibrillation, history of myocardial infarction or heart failure, (supra)-ventricular tachycardia, atrioventricular nodal re-entrant tachycardia, second or third degree atrioventricular block, bundle branch block and use of a pacemaker. Furthermore, we excluded individuals on heart rate altering medications (non-dihydropyridine calcium antagonists (Anatomic Therapeutic Chemical (ATC) code C08D, digoxin (ATC code C01AA5), and amiodarone (ATC code C01BD01)) as these could affect QT dynamics.

We also excluded individuals with an extreme heart rate during rest (<40 or >120 bpm), peak exercise (>200 bpm) or at 1 min post-exercise (<40 or >200 bpm), individuals with very small changes in RR interval (inverse of heart rate) from rest to exercise, or exercise to recovery was (ΔRR <10 ms), and individuals with poor quality ECG recording.

Genome wide association analyses

Following genetic and phenotypic quality control, 51,884 and 51,503 individuals of European ancestry from the exercise cohort were included in the genome wide association analyses for QT dynamics during exercise and recovery (Figure 1). We applied inverse-normal transformations before genetic analyses to correct for skewed distributions of the QT dynamics markers (Supplemental Fig. 3). Model SNVs were selected from the genotyped SNVs, required for the subsequent GWASs using PLINK 1.9[7]. This selection was based on the following criteria: a minor allele frequency (MAF) > 1%, a Hardy-Weinberg equilibrium (HWE) with a threshold of P-value = $1x10^{-6}$, and missingness < 0.0015. Using the model SNVs and ~ 9 million imputed variants with MAF ≥ 1%, and imputation quality (INFO) > 0.3, heritability was calculated for each phenotype in the full datasets using a variance components method with BOLT-REML[8]. GWASs were performed to discover SNVs associated with QT dynamics during exercise and recovery using a linear mixed model method (BOLT-LMM)[8] under the additive genetic model including ~ 9 million imputed SNVs with MAF ≥ 1% and INFO > 0.3. We included sex, diabetes, age, body mass index (BMI), genotyping array (binary indicator: UKB vs. UK BiLEVE) as covariates. In addition, we also included trait-specific covariates: RR_{rest} and ΔRR_{ex} for the QT dynamics during exercise trait; RR_{rec} and ΔRR_{rec} for the QT during dynamics recovery trait.

Since we did not have access to an independent study that could serve as a replication study, we randomly divided our dataset into discovery (N ~ 30,000) and replication (N ~ 22,000) datasets and removed individuals with kinship coefficient > 0.088[9]. We compiled all SNVs with $P < 1 \times 10^{-6}$ from the discovery analysis and mapped them to individual loci based on genomic distance of > 500 Kb to each side of the lead SNV. If multiple SNVs fit the selection criteria for a single region, only the SNV with the smallest P value was considered for follow up. As a QC step, for each trait we reviewed the selected SNVs to check for unrealistically high effect sizes, large standard errors, and none were observed. Regional association plots were produced for all selected SNVs and these were carefully reviewed. For each trait,

replication was confirmed if the P-value in the replication cohort was lower than the Bonferroni threshold and the effect size was in the same direction observed in discovery analyses in the replication cohort.

In addition to the replication analyses, we also performed a full dataset GWAS for the three traits. Additional loci, considering one lead SNV per 1 Mb region, for each trait reaching a genome-wide significance threshold ($P \leq 5 \times 10^{-8}$) from the full dataset GWAS were identified. In addition to the replication study, we also performed a GWAS in the full datasets and sex-stratified GWASs to identify additional loci.

Conditional analysis

For each trait, we examined the existence of SNVs independent to lead SNVs but tagging the same loci by applying genome-wide complex trait analysis (GCTA)[10] for all validated and genome-wide significant loci from the full dataset GWAS. A secondary signal would be declared if: (i) the newly identified SNV original *P* value was lower than 1×10^{-6}; (ii) there was less than a 1.5-fold difference between the lead SNV and secondary association *P* values on a $-\log_{10}$ scale, i.e., if $-\log_{10}(P_{lead})/-\log_{10}(P_{\mathrm{sec}}) < 1.5$; and (iii) there was less than a 1.5-fold difference between the main association and conditional association *P* values on a $-\log_{10}$ scale, i.e., if $-\log_{10}(P_{sec})/-\log_{10}(P_{\mathrm{cond}}) < 1.5$.

Percent variance

For each trait, the percent of variance explained by all genome-wide significant variants and the secondary signals was derived by generating the residuals from the regression model of each trait against the covariates used in each respective genetic model. We then fitted a second linear model for the trait residuals with all the identified variants plus the top ten principal components to assess the variance explained.

Sex-stratified analyses

We performed sex-stratified analyses in the full cohort (QT dynamics during exercise: 28,449 females and 24,412 males; QT dynamics during recovery: 28,689 females, 24,172 males) including the same covariates in the regression model as the full dataset analyses but excluding sex. We then looked for genetic variants that were significantly associated with any of the traits in one of the sex-specific cohorts, but not in the primary analysis.

Overlap with reported loci for resting QT

To determine whether loci overlapped with resting QT interval, we downloaded all reported SNVs for resting QT from the NHGRI GWAS catalog. At the time of writing, the catalogue had not been updated with the latest results from Bihlmayer et al.[11] and Van Setten et al.[12], which we added to the list resulting in 43 loci in total by mapping individual loci based on genomic distance of > 500 Kb to each side of the reported SNVs.. We then filtered out variants that were not genome or exome wide significant ($P < 5 \times 10^{-8}$ or $P < 2 \times 10^{-7}$, respectively). Then for each known variant, we calculated the pairwise LD for all these variants within a 4Mb region (± 2Mb) around each known variant using PLINK v2[7]. Next, the multiple variants in LD ($r^2 > 0.1$) with each known variant were ordered according to their positions on the chromosome, and we defined a window with the start position of the first variant in the ordered list and the end position of the last variant in the ordered list. The start and end of the window was extended by 50kb on either side. This LD defined window or a window of ±500kb, which ever was the larger, was considered as the known locus.

Bioinformatics analyses

We performed comprehensive bioinformatics analyses to annotate loci at both variant (all SNVs in linkage disequilibrium (LD), $r^2 \geq 0.8$, were considered) and gene level. The LD was calculated using genetic data from UKB within a 8Mb region (± 4Mb) around each known variant using PLINK v2[7]. For each lead SNV, we annotated the nearest genes and genes in which SNVs in LD ($r^2 > 0.4$) with the lead SNV are located using University of California, Santa Cruz (UCSC) known genes. Variant effect predictor (VEP) analyses determined the effect of

the variants, including the impact of amino acid substitutions[13]. We also investigated whether any of the discovered GWAS signals co-localized with genetic variants that regulate expression in adrenal, heart and brain tissues using expression quantitative trait locus (eQTL) signals from the Genotype-Tissue Expression (GTEx) dataset version 7[14] using COLOC software[15]. Potential target genes of regulatory SNVs were identified using long-range chromatin interaction (Hi–C) data[16]. In addition, DEPICT[17] (Data-driven Expression-Prioritised Integration for Complex Traits) analyses prioritized likely causal genes and tissues. Enrichments with FDR < 0.05 were deemed significant. A literature review examined all identified genes at a locus in order to prioritize which candidate genes were used as an input for g:profiler[18]. Finally, trait pleiotropy was assessed by querying associated SNVs for other traits using PhenoScanner[19]. We also queried gene-specific animal models using International Mouse Phenotyping Consortium and the Mouse Genome Informatics database[20].

Survival analyses

We conducted a survival analyses to investigate the prognostic value of QT dynamics during exercise and recovery in 56,643 individuals without a history of CV disease (Figure 1). The following covariates were included: age, sex, diabetes, high cholesterol, body mass index (BMI), systolic blood pressure (SBP), diastolic blood pressure (DBP), resting heart rate, heart rate increase/decrease during exercise and recovery, and the resting heart rate-corrected QT (QTc) interval. The prognostic value was investigated for two endpoints: (1) CV event (death or hospitalization), and (2) all-cause mortality (ACM). A CV event was defined as either myocardial infarction, ischemic heart disease, cardiac arrest, heart failure, or stroke. Follow-up data was available from the Hospital Episode Statistics (HES) and national death registry data provided by UKB (coded in the 10th revision of the International Statistical Classification of Diseases and Related Health Problems). We used both main and secondary diagnoses. Date of an event was defined as the date of the first diagnosis. Follow-up was available from inclusion until 31 March 2017. A list of the *ICD-10* codes is provided in Supplemental Table 2. Two-tailed Mann-Whitney or Chi-squared tests were used to evaluate which variables were

significantly different for individuals reaching the endpoints. Variables for which differences were statistically significant ($P < 0.05$) were included in univariate and, if differences remained significant, in the multivariate Cox regression analyses to investigate their predictive value. Continuous variables were standardized to have a mean of 0 and a standard deviation of 1. Proportional hazards assumptions were tested using Schoenfeld residuals. A P-value of < 0.05 was considered statistically significant. Statistical analyses were performed with R version 3.5.1 using the *survival* package.

Genetic risk scores

To investigate whether the genetic variants associated with QT dynamics during exercise and recovery modulate CV risk in healthy individuals, we constructed weighted genetic risk scores (GRSs) using all identified lead and secondary SNVs. Beta coefficients from the replication analyses were used as weights. We next evaluated whether the GRSs were associated with CV risk in 357,822 unrelated individuals from UKB comprised of European ancestry who were not included in the GWAS, passed genetic QC, and were free of a previous history of CV events (Figure 1). The impact of the GRS was evaluated by comparing the incidence of CV events in the top 5% vs bottom 5% of the GRS distribution using the Chi-square test.

References

1. Sudlow C, Gallacher J, Allen N, Beral V, Burton P, Danesh J, Downey P, Elliott P, Green J, Landray M, et al. Uk biobank: An open access resource for identifying the causes of a wide range of complex diseases of middle and old age. *PLOS Medicine*. 2015;12:e1001779
2. Pilhall M, Riha M, Jern S. Exercise-induced qrs changes in healthy men and women: A multivariate analysis on their relation to background data and exercise performance. *European heart journal*. 1992;13:1316-1324
3. Porta A, Girardengo G, Bari V, George AL, Brink PA, Goosen A, Crotti L, Schwartz PJ. Autonomic control of heart rate and qt interval variability influences arrhythmic risk in long qt syndrome type 1. *Journal of the American College of Cardiology*. 2015;65:367-374

4. Ramírez J, van Duijvenboden S, Aung N, Laguna P, Pueyo E, Tinker A, Lambiase PD, Orini M, Munroe PB. Cardiovascular predictive value and genetic basis of ventricular repolarization dynamics. *Circulation: Arrhythmia and Electrophysiology*. 2019;12:e007549
5. Lepeschkin E, Surawicz B. The measurement of the qt interval of the electrocardiogram. *Circulation*. 1952;6:378-388
6. Bycroft C, Freeman C, Petkova D, Band G, Elliott LT, Sharp K, Motyer A, Vukcevic D, Delaneau O, O'Connell J. The uk biobank resource with deep phenotyping and genomic data. *Nature*. 2018;562:203-209
7. Chang CC, Chow CC, Tellier LC, Vattikuti S, Purcell SM, Lee JJ. Second-generation plink: Rising to the challenge of larger and richer datasets. *GigaScience*. 2015;4:7
8. Loh P-R, Bhatia G, Gusev A, Finucane HK, Bulik-Sullivan BK, Pollack SJ, Schizophrenia Working Group of the Psychiatric Genomics C, de Candia TR, Lee SH, Wray NR, et al. Contrasting genetic architectures of schizophrenia and other complex diseases using fast variance-components analysis. *Nature Genetics*. 2015;47:1385
9. Bycroft C, Freeman C, Petkova D, Band G, Elliott LT, Sharp K, Motyer A, Vukcevic D, Delaneau O, O'Connell J, et al. Genome-wide genetic data on ~500,000 uk biobank participants. *bioRxiv*. 2017
10. Yang J, Lee SH, Goddard ME, Visscher PM. Gcta: A tool for genome-wide complex trait analysis. *The American Journal of Human Genetics*. 2011;88:76-82
11. Bihlmeyer NA, Brody JA, Smith AV, Warren HR, Lin H, Isaacs A, Liu C-T, Marten J, Radmanesh F, Hall LM. Exomechip-wide analysis of 95 626 individuals identifies 10 novel loci associated with qt and jt intervals. *Circ Genom Precis Med*. 2018;11:e001758
12. van Setten J, Verweij N, Mbarek H, Niemeijer MN, Trompet S, Arking DE, Brody JA, Gandin I, Grarup N, Hall LM. Genome-wide association meta-analysis of 30,000 samples identifies seven novel loci for quantitative ecg traits. *European Journal of Human Genetics*. 2019;27:952
13. McLaren W, Gil L, Hunt SE, Riat HS, Ritchie GR, Thormann A, Flicek P, Cunningham F. The ensembl variant effect predictor. *Genome biology*. 2016;17:122
14. Consortium G. Genetic effects on gene expression across human tissues. *Nature*. 2017;550:204
15. Giambartolomei C, Vukcevic D, Schadt EE, Franke L, Hingorani AD, Wallace C, Plagnol V. Bayesian test for colocalisation between pairs of genetic association studies using summary statistics. *PLoS genetics*. 2014;10:e1004383
16. Schmitt AD, Hu M, Jung I, Xu Z, Qiu Y, Tan CL, Li Y, Lin S, Lin Y, Barr CL. A compendium of chromatin contact maps reveals spatially active regions in the human genome. *Cell reports*. 2016;17:2042-2059
17. Pers TH, Karjalainen JM, Chan Y, Westra H-J, Wood AR, Yang J, Lui JC, Vedantam S, Gustafsson S, Esko T, et al. Biological interpretation of genome-wide association studies using predicted gene functions. *Nature Communications*. 2015;6:5890
18. Raudvere U, Kolberg L, Kuzmin I, Arak T, Adler P, Peterson H, Vilo JJNar. G: Profiler: A web server for functional enrichment analysis and conversions of gene lists (2019 update). 2019
19. Staley JR, Blackshaw J, Kamat MA, Ellis S, Surendran P, Sun BB, Paul DS, Freitag D, Burgess S, Danesh J. Phenoscanner: A database of human genotype–phenotype associations. *Bioinformatics*. 2016;32:3207-3209
20. Bult CJ, Blake JA, Smith CL, Kadin JA, Richardson JE. Mouse genome database (mgd) 2019. *Nucleic acids research*. 2018;47:D801-D806

Supplemental Figures

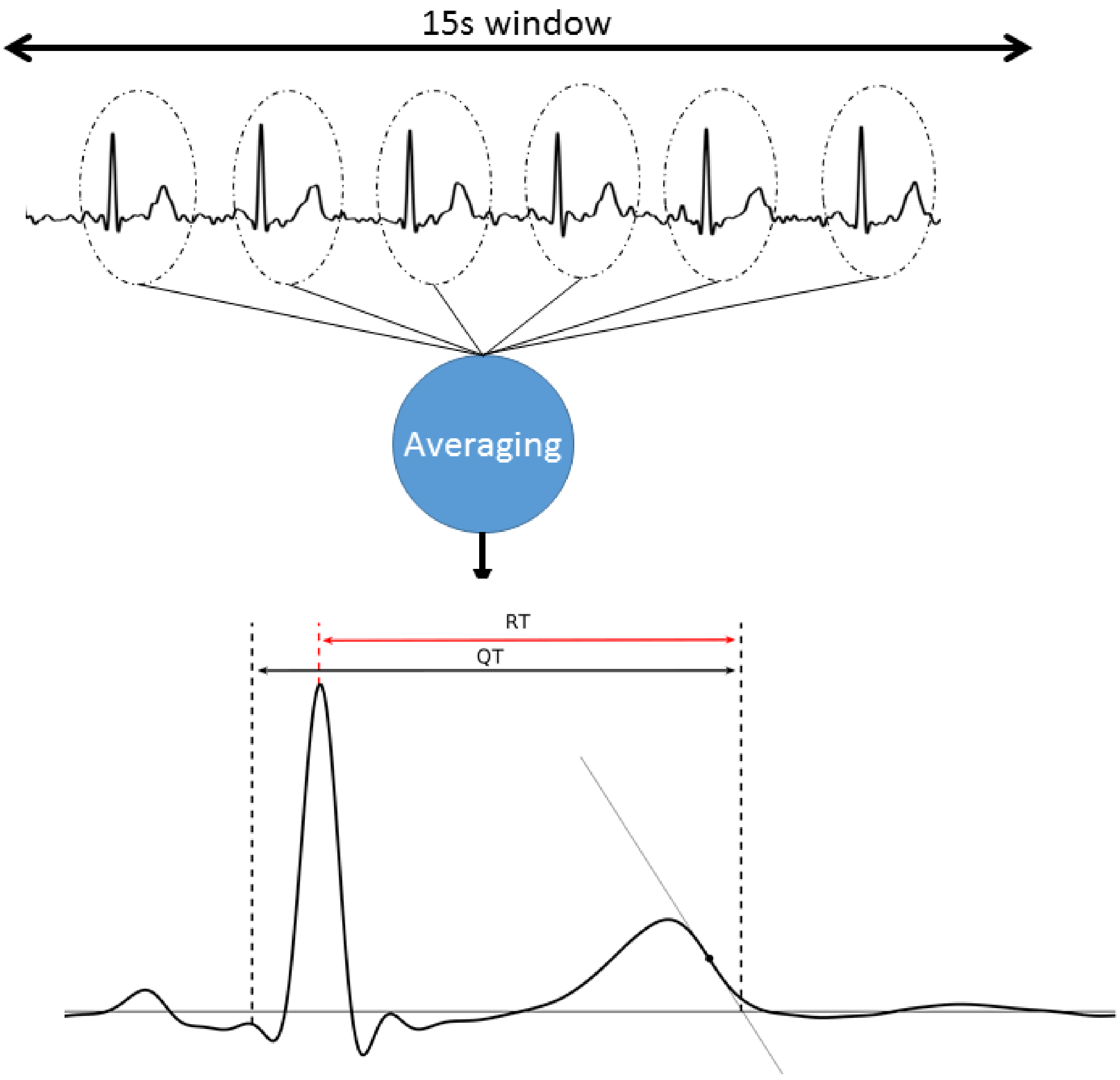

Supplemental Figure 1. ***Derivation of average heart beat and QRST-waveform.***

The end of the T-wave (Tend) was measured using the Tangent method. The QT interval was measured between QRS onset and the end of the T-wave. The RT interval was measured from R-peak to the end of the T-wave.

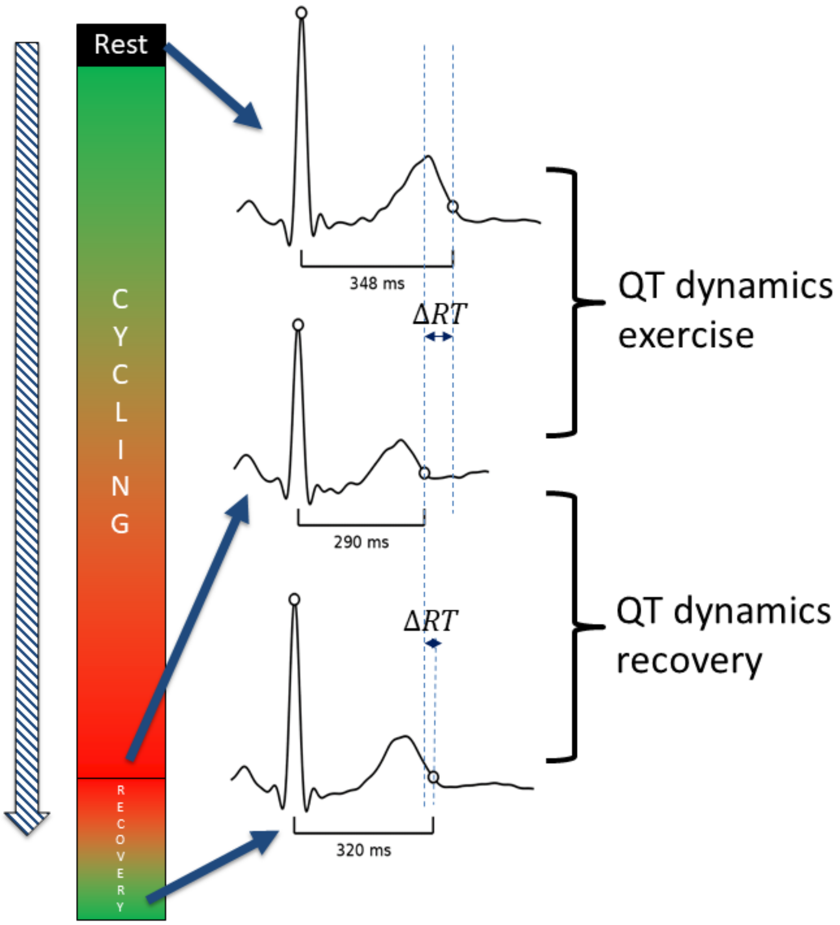

Supplemental Figure 2: ***Schematic illustration of the QT dynamics measurements during exercise and recovery.***

Signal-averaged ECGs were obtained at three timepoints during the exercise protocol: rest (pre-exercise), peak-exercise, and recovery. At each time point, we computed the signals averaged ECG waveform to measure the RT interval. The QT dynamics during exercise was then approximated by the change in RT interval between rest and peak-exercise divided by the corresponding change in the RR interval. Similarly, the QT dynamics during recovery was approximated by dividing the change in RT interval between recovery and peak-exercise by the corresponding change in the RR interval. The RR interval corresponded to the median RR interval of all heart beats used to compute the averaged ECG waveform.

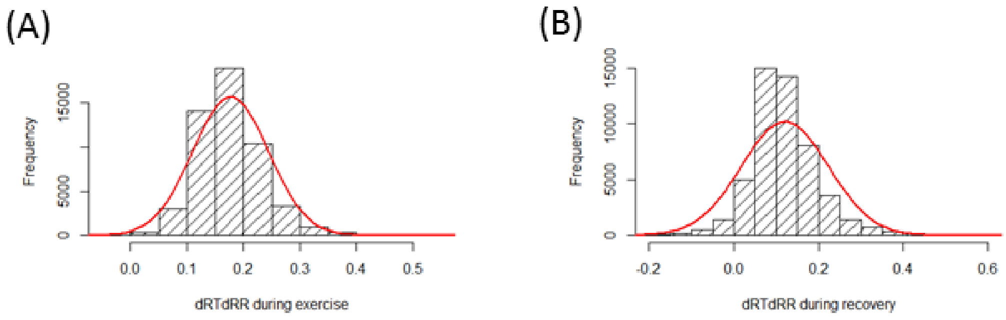

Supplemental Figure 3. ***Histograms of QT dynamics during exercise (A) and QT dynamics during recovery (B).***

The red line shows the estimated distribution shape.

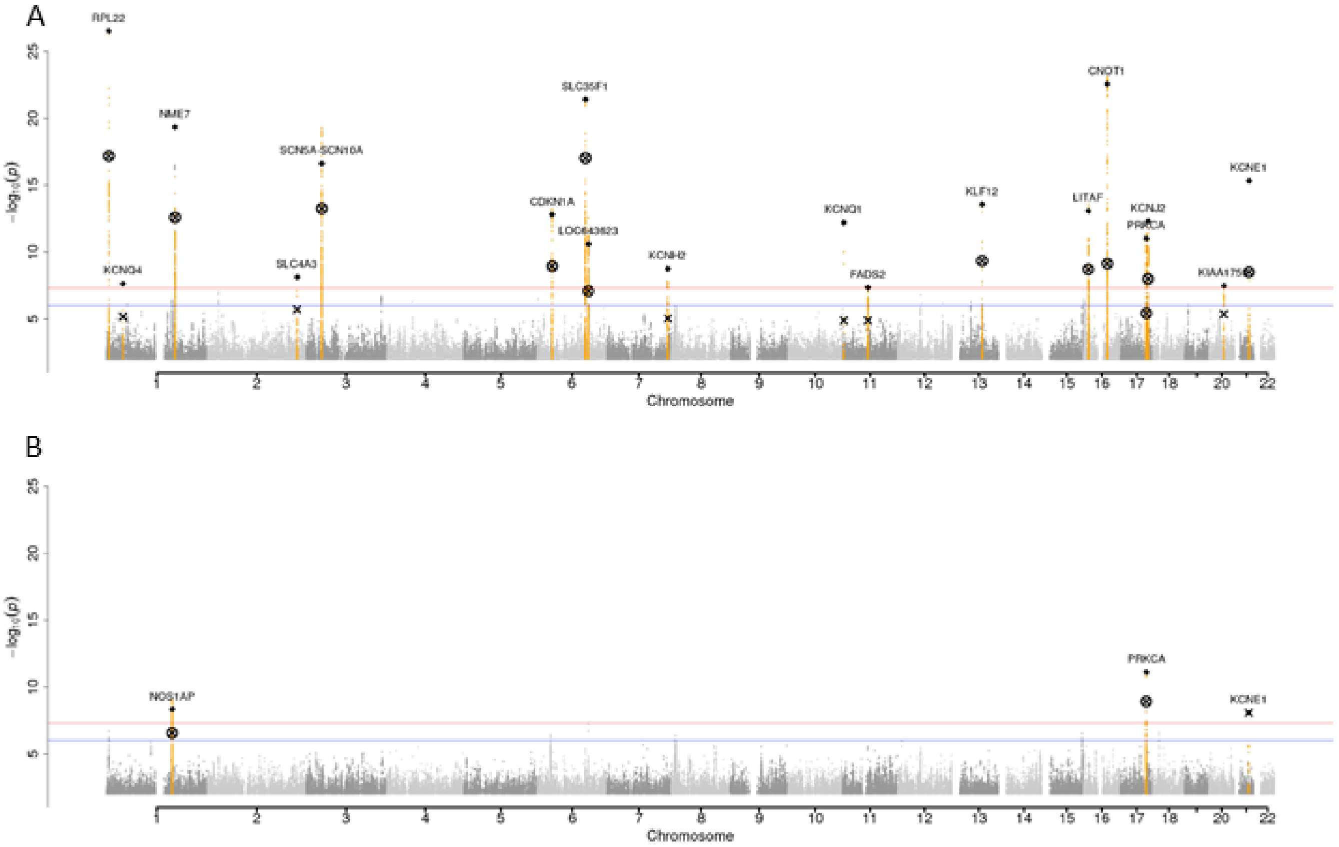

Supplemental Figure 4. ***Association results of the QT dynamics GWAS in the full data.***

Manhattan plot from the full GWAS for QT dynamics during exercise (A) and recovery (B) in full data. P values, expressed as −log10(P), are plotted according to physical genomic locations by chromosome. Genome-wide significant loci are highlighted in yellow. Lead SNVs are marked by the triangles including all novel loci (yellow). The crosses indicate the P values of these SNVs in the discovery dataset. Crosses are encircled for SNPs that formally replicated. Locus names of the novel loci correspond to the nearest annotated gene. The blue horizontal line indicates a P value threshold of 1×10^{-6}, corresponding to the lookup significance threshold in the discovery analysis. The red horizontal line indicates a P-value threshold of 5×10^{-8}, corresponding to genome-wide significance.

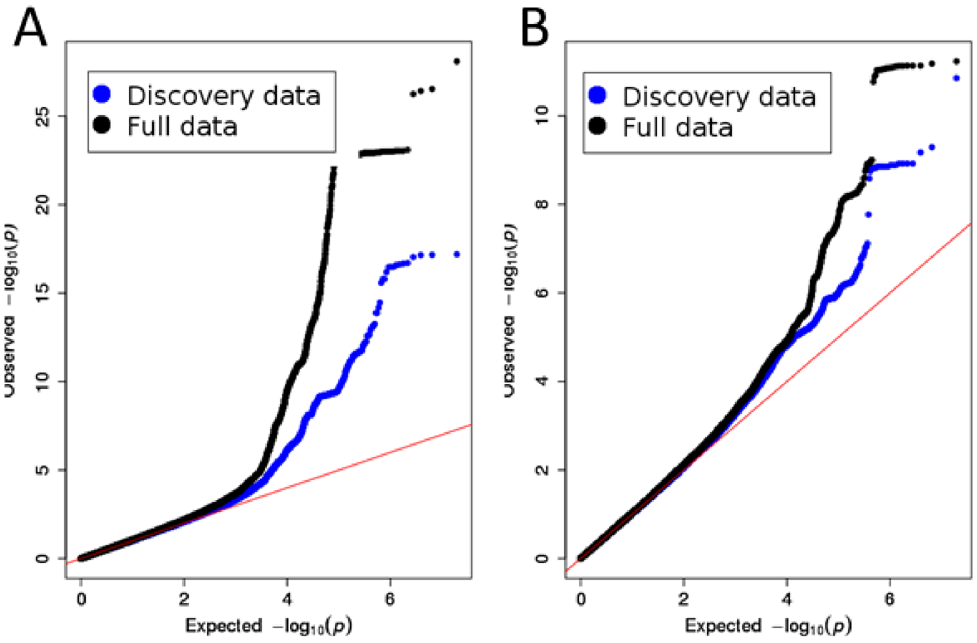

Supplemental Figure 5. ***QQ plots QT dynamics during exercise (A), and recovery (B).***

Discovery data (blue) and full cohort analysis (black) data. Corresponding λ values are 1.05 and 1.05 for QT dynamics during exercise, and 1.002 and 1.05 for QT dynamics during recovery.

rs10864434

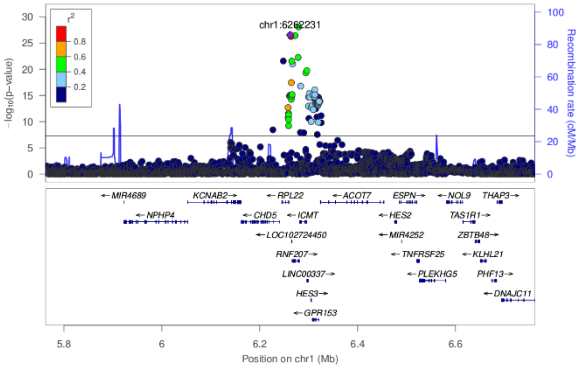

rs116015634

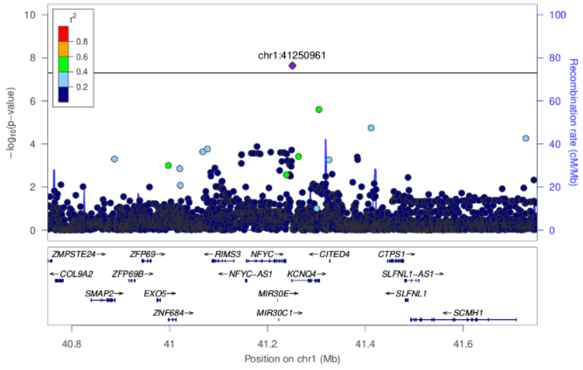

rs1591734

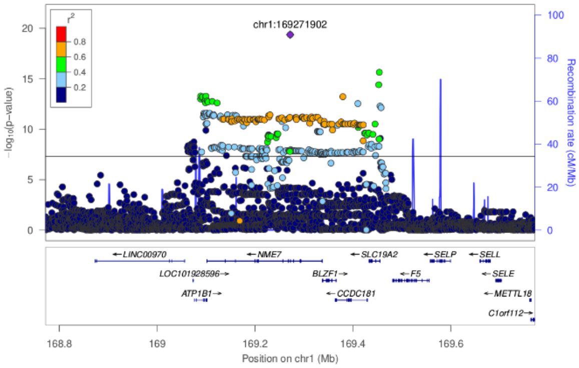

rs35394392

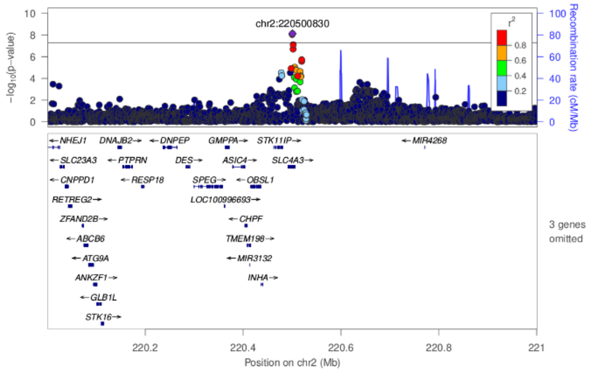

rs7638275

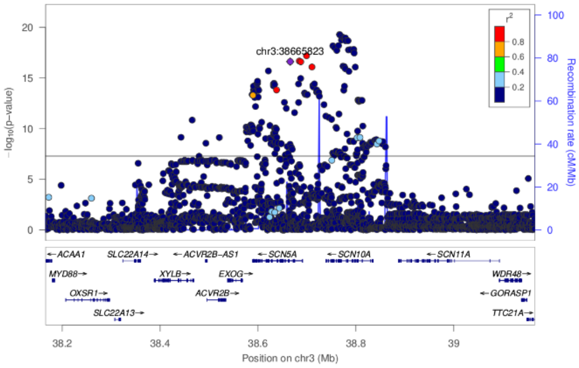

rs1321311

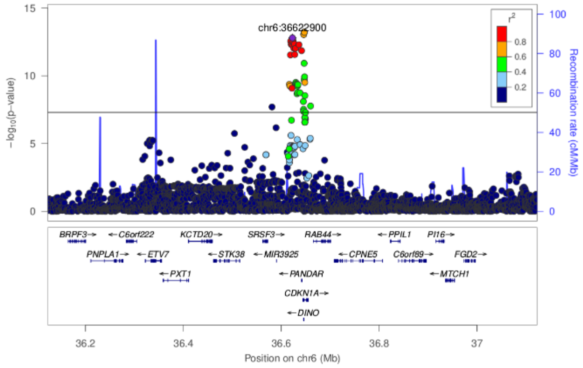

rs28436726

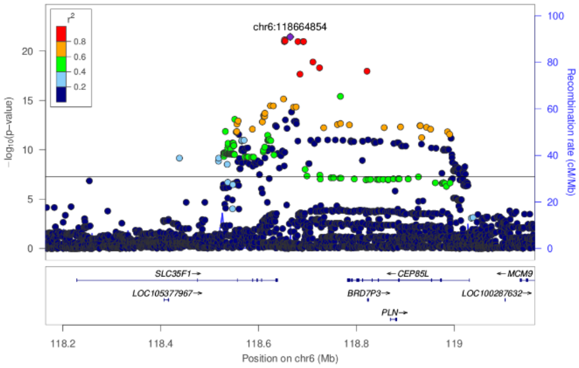

rs35846768

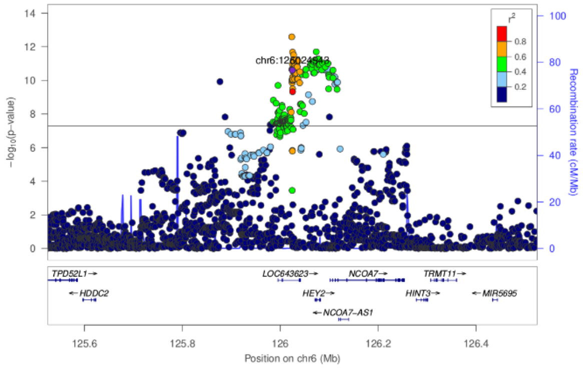

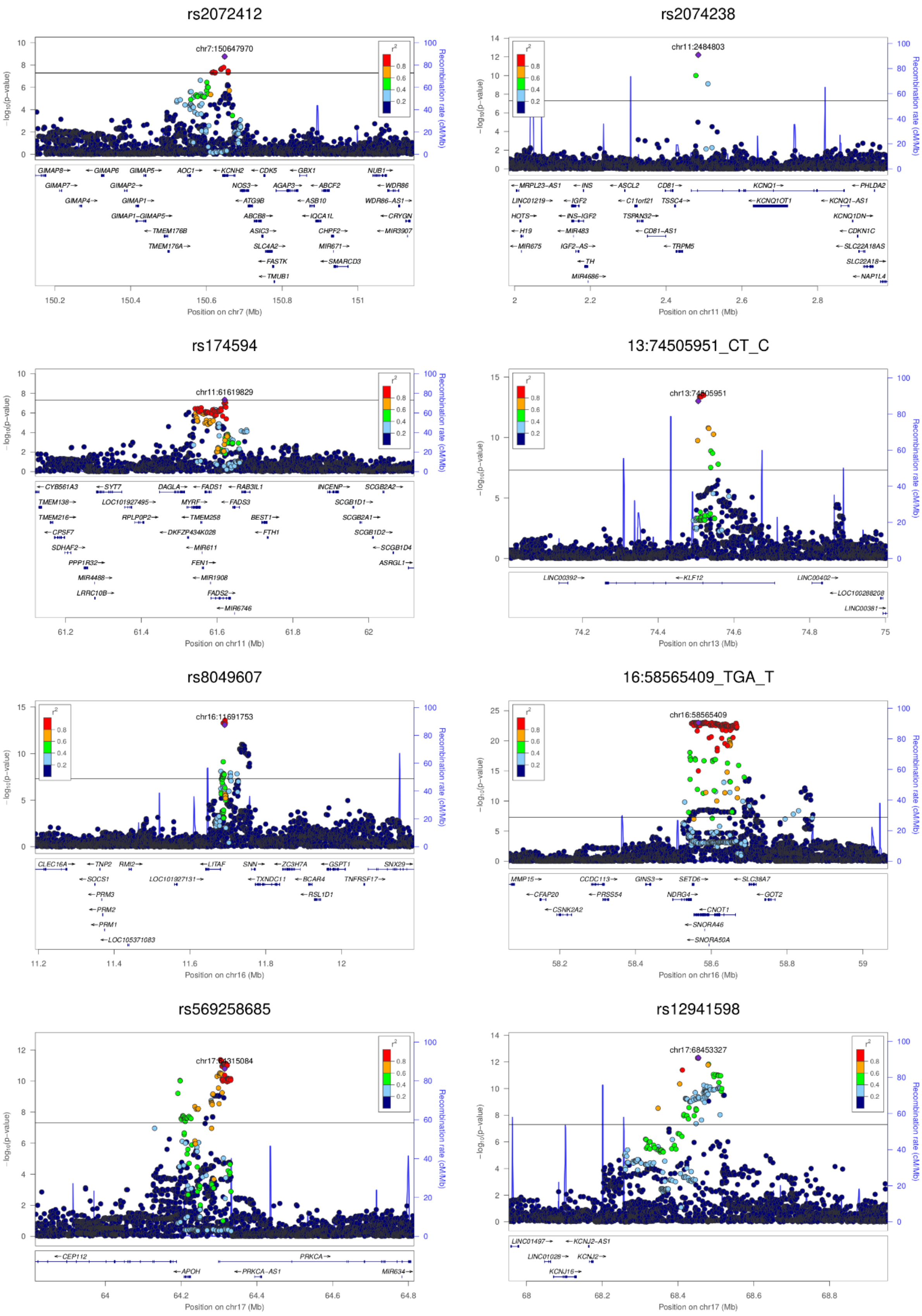
rs2072412
chr7:150647970
−log10(p−value)
Recombination rate (cM/Mb)
GIMAP8 GIMAP6 GIMAP5 AOC1 KCNH2 CDK5 GBX1 NUB1
GIMAP7 GIMAP2 NOS3 AGAP3 ABCF2 WDR86
GIMAP4 GIMAP1 ATG9B ASB10 WDR86−AS1
GIMAP1−GIMAP5 ABCB8 IQCA1L CRYGN
TMEM176B ASIC3 CHPF2 MIR3907
TMEM176A SLC4A2 MIR671
FASTK SMARCD3
TMUB1
Position on chr7 (Mb)
rs2074238
chr11:2484803
MRPL23−AS1 INS ASCL2 CD81 KCNQ1 PHLDA2
LINC01219 IGF2 C11orf21 TSSC4 KCNQ1OT1 KCNQ1−AS1
HOTS INS−IGF2 TSPAN32 KCNQ1DN
H19 MIR483 CD81−AS1 CDKN1C
MIR675 IGF2−AS TRPM5 SLC22A18AS
TH SLC22A18
MIR4686 NAP1L4
Position on chr11 (Mb)
rs174594
chr11:61619829
CYB561A3 SYT7 DAGLA FADS1 RAB3IL1 INCENP SCGB2A2
TMEM138 LOC101927495 MYRF FADS3 SCGB1D1
TMEM216 RPLP0P2 TMEM258 BEST1 SCGB2A1
CPSF7 DKFZP434K028 FTH1 SCGB1D2
SDHAF2 MIR611 SCGB1D4
PPP1R32 FEN1 ASRGL1
MIR4488 MIR1908
LRRC10B FADS2
MIR6746
Position on chr11 (Mb)
13:74505951_CT_C
chr13:74505951
LINC00392 KLF12 LINC00402
LOC100288208
LINC00381
Position on chr13 (Mb)
rs8049607
chr16:11691753
CLEC16A TNP2 RMI2 LITAF SNN ZC3H7A GSPT1 SNX29
SOCS1 LOC101927131 TXNDC11 BCAR4 TNFRSF17
PRM3 RSL1D1
PRM2
PRM1
LOC105371083
Position on chr16 (Mb)
16:58565409_TGA_T
chr16:58565409
MMP15 CCDC113 GINS3 SETD6 SLC38A7
CFAP20 PRSS54 NDRG4 GOT2
CSNK2A2 CNOT1
SNORA46
SNORA50A
Position on chr16 (Mb)
rs569258685
chr17:64315084
CEP112 PRKCA
APOH PRKCA−AS1 MIR634
Position on chr17 (Mb)
rs12941598
chr17:68453327
LINC01497 KCNJ2−AS1
LINC01028 KCNJ2
KCNJ16
Position on chr17 (Mb)

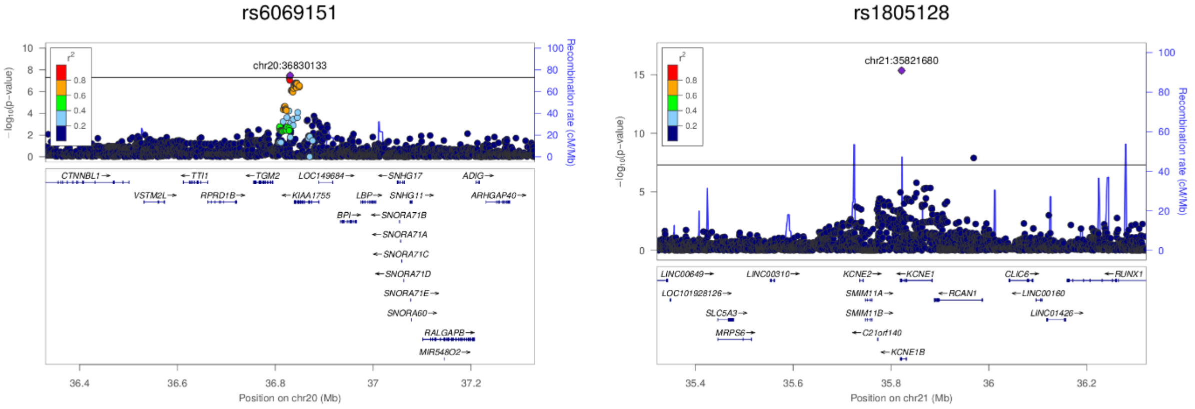

Supplemental Figure 6A. ***Regional plots for all lead variants for QT dynamics during exercise in the full analysis.***

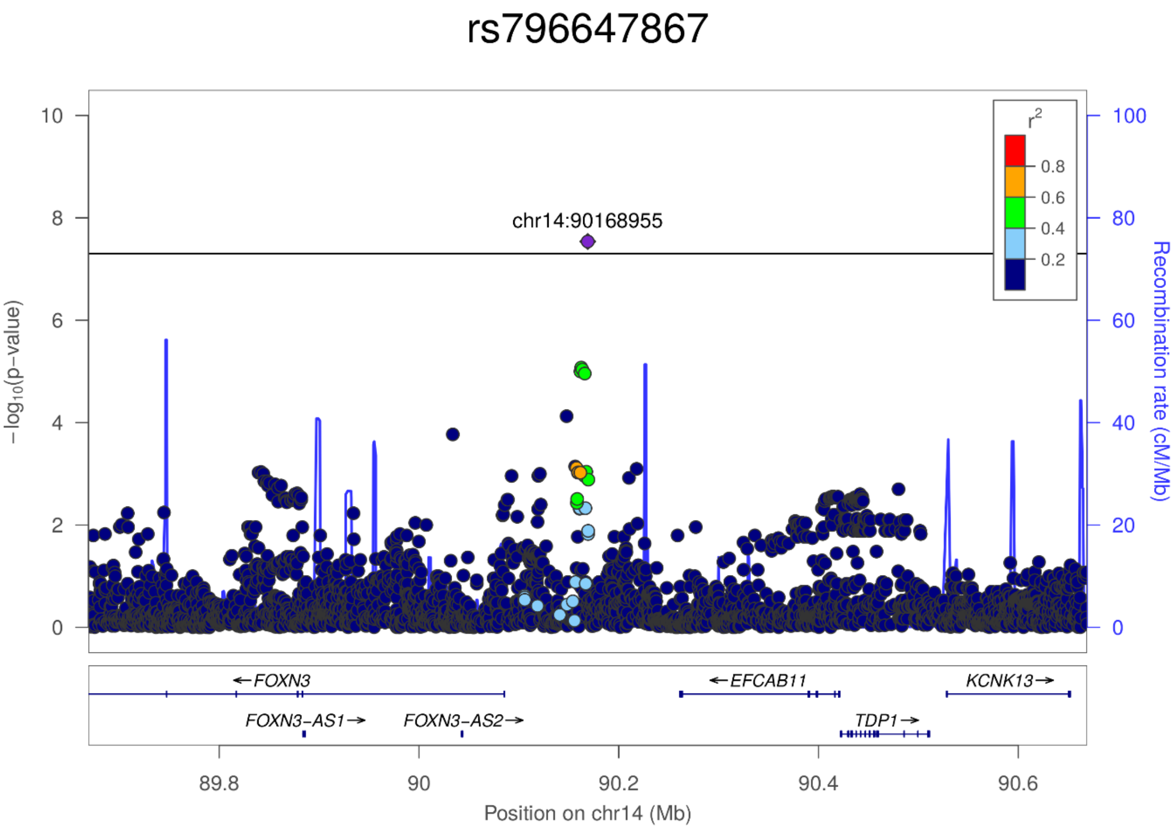

Supplemental Figure 6B. ***Regional plot for the sex-specific lead variant for QT dynamics during exercise in the full analysis of male data.***

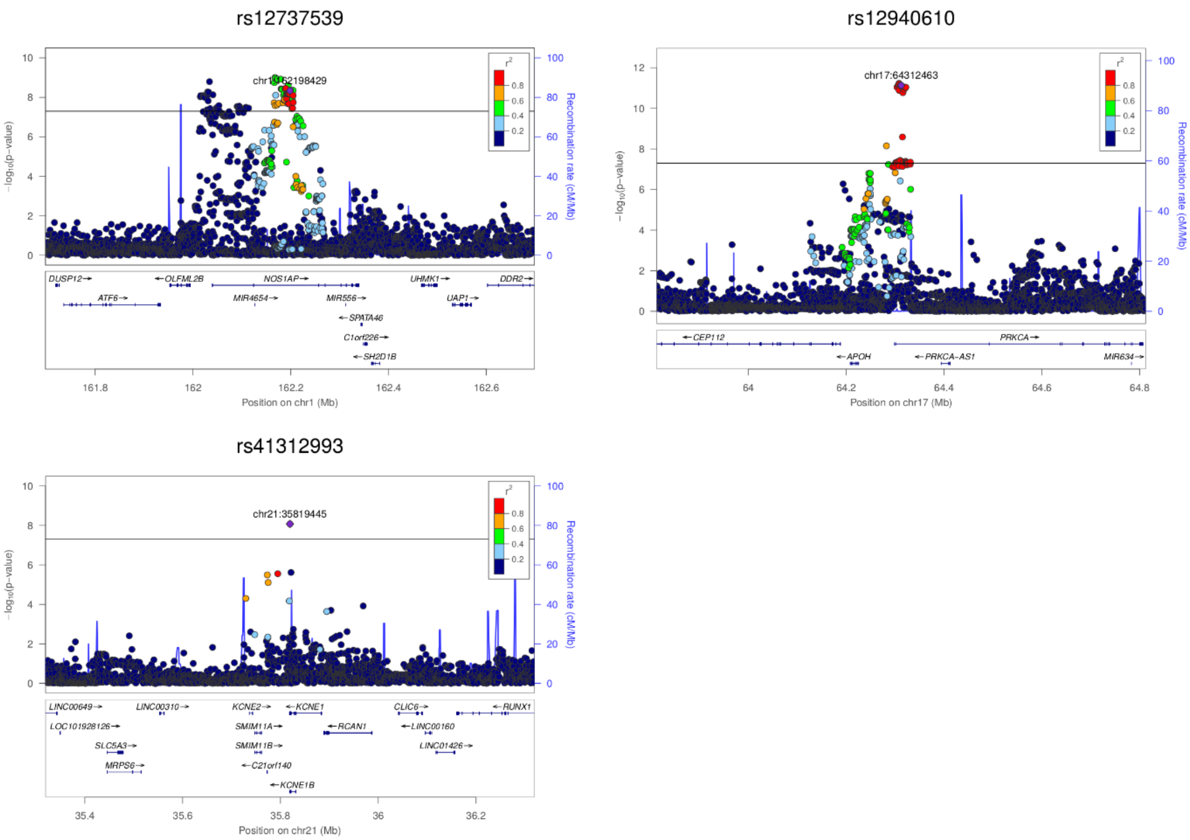

Supplemental Figure 6C. ***Regional plots for all lead variants for QT dynamics during recovery in the full analysis.***

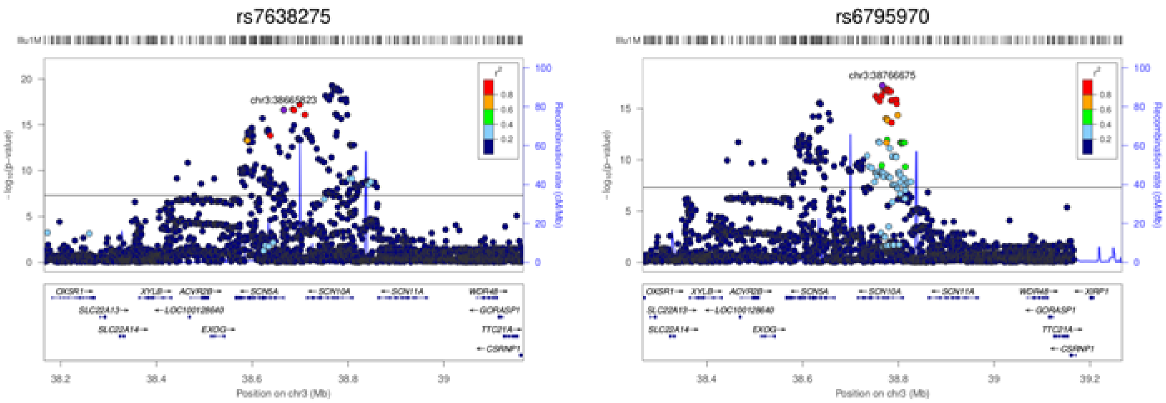

Supplemental Figure 7A. ***Regional plots lead signal and secondary signal at SCN5A-SCN10A locus for QT dynamics during exercise***

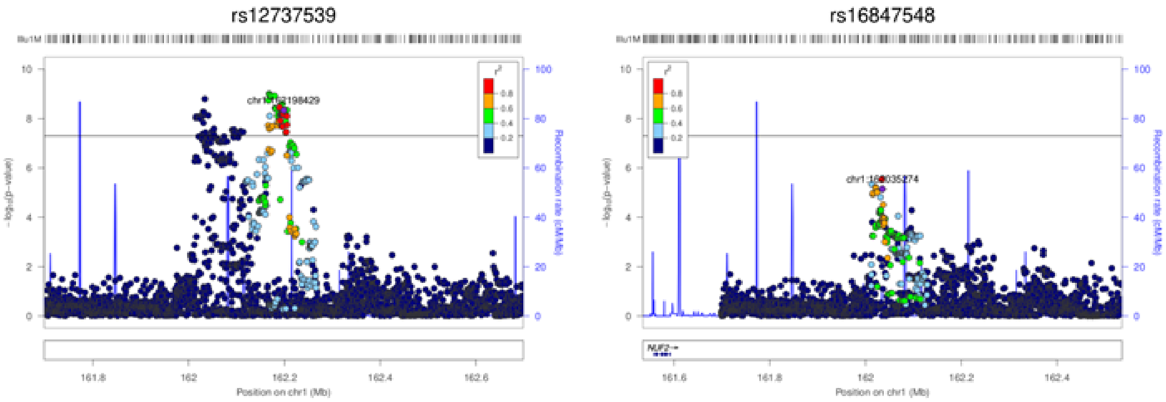

Supplemental Figure 7B. ***Regional plots lead signal and secondary signal at NOS1AP locus for QT dynamics during recovery***

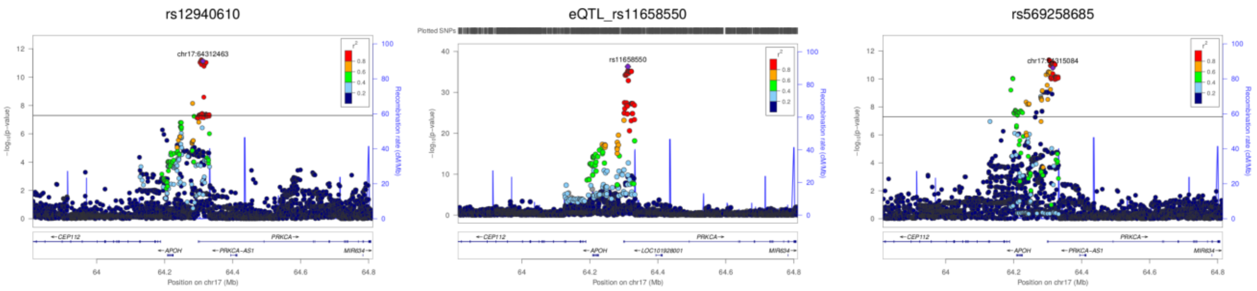

Supplemental Figure 8: ***Regional plots showing colocalization of QT dynamics GWASs and eQTL gene expression signals at PRKCA locus.***

Left panel: GWAS signal for QT dynamics during recovery, middle: gene expression eQTL signal of PRKCA for left ventricular tissue, right: GWAS signal for QT dynamics during recovery. Posterior probabilities for a common signal between QT dynamics and the eQTL signal where 81 and 85% for exercise and recovery respectively

Supplemental Tables: see separate Excel (.xlsx) file

Supplemental Table 1. Demographics of the discovery, replication and full datasets.
Supplemental Table 2. ICD-10 codes used in follow-up analysis
Supplemental Table 3. Sex-stratified analyses for QT dynamics during exercise
Supplemental Table 4. Overlap between loci associated with QT dynamics during exercise and recovery, and resting QT interval.
Supplemental Table 5. Phenoscanner lookup for novel variants associated with QT dynamics during exercise from loci not overlapping with QT interval. Variants reported by from peer-reviewed journals (A) and from Neale's lab (B).
Supplemental Table 6A. Annotation of lead and conditionally independent variants and their proxies (r2>0.8) of QT dynamics during exercise loci using Variant Effect Predictor.
Supplemental Table 6B. Annotation of lead and conditionally independent variants and their proxies (r2>0.8) of QT dynamics during recovery loci using Variant Effect Predictor.
Supplemental Figure 7. Chromatin interaction (Hi-C) results for all lead and conditionally independent variants or their proxies (r2>0.8) for QT dynamics loci.
Supplemental Figure 8. Significant cis-eQTLs for QT dynamics associated variants
Supplemental Figure 9A. DEPICT tissue and cell type enrichment results across QT dynamics during exercise
Supplemental Figure 9B. DEPICT gene prioritization results for QT dynamics during exercise
Supplemental Figure 10. Candidate genes at loci associated with QT dynamics during exercise and recovery
Supplemental Figure 11A. g:profiler results for QT dynamics during exercise. Input genes are the candidate genes for QT dynamics during exercise highlighted in Supplementary Table 11.
Supplemental Figure 11B. g:profiler results for QT dynamics during recovery. Input genes are the candidate genes for QT dynamics during exercise highlighted in Supplementary Table 11.
