# Supplementary material for: Genetic Basis and Prognostic Value of Exercise QT Dynamics
Source: Circ Genom Precis Med. 2020 Jun 11;13(4):e002774. doi: 10.1161/CIRCGEN.119.002774 (PMC7439940; doi:10.1161/CIRCGEN.119.002774)
Supplement: Supplementary file 3 [file hcg-13-e002774-s003.pdf]

Genetic Basis and Prognostic Value of Exercise QT Dynamics

Running title: *Duijvenboden & Ramírez et al.; Genetic Basis and Prognostic Value of QT Dynamics*

Stefan van Duijvenboden, PhD[1,2]*; Julia Ramírez, PhD[1,2]*; William J. Young, MD[2,3];

Borbala Mifsud, PhD[2,4]; Michele Orini, PhD[1,2]†; Andrew Tinker, MD, PhD[2,5]†;

Patricia B. Munroe, PhD[2,5]*†; Pier D. Lambiase, MD, PhD[1,3]*†

[1]Institute of Cardiovascular Science, University College London; [2]Clinical Pharmacology, William Harvey Research Institute, [5]NIHR Barts Cardiovascular Biomedical Research Unit, Barts & The London School of Medicine and Dentistry, Queen Mary University of London; [3]Barts Heart Centre, St Bartholomew's Hospital, London, United Kingdom; [4]College of Health and Life Sciences, Doha, Qatar
*contributed equally / †joint supervisors

Circulation: Genomic and Precision Medicine

Correspondence:

Professor Patricia B. Munroe
Clinical Pharmacology,
William Harvey Research Institute,
Barts & The London School of Medicine and Dentistry,
Queen Mary University of London,
London, EC1M 6BQ
United Kingdom
E-mail: p.b.munroe@qmul.ac.uk

Professor Pier D. Lambiase
Institute of Cardiovascular Science,
University College London,
London, WC1E 6BT,
United Kingdom
E-mail: p.lambiase@ucl.ac.uk

Journal Subject Terms: Electrophysiology; Autonomic Nervous System; Genetic, Association Studies; Cardiovascular Disease; Exercise

Abstract:

Background - Abnormal QT interval responses to heart rate (QT dynamics) is an independent risk predictor for cardiovascular disease in patients, but its genetic basis and prognostic value in a population-based cohort have not been investigated.

Methods - QT dynamics during exercise and recovery were derived in 56,643 individuals from UK Biobank without a history of cardiovascular events. Genome-wide association studies (GWAS) were conducted to identify genetic variants and bioinformatics analyses were performed to prioritize candidate genes. The prognostic value of QT dynamics was evaluated for cardiovascular events (death or hospitalization) and all-cause mortality.

Results - Heritability of QT dynamics during exercise and recovery were 10.7% and 5.4% respectively GWASs identified 20 loci, of which four loci included genes implicated in mendelian long QT syndrome. Five loci did not overlap with previously reported resting QT interval loci, candidate genes included *KCNQ4* and *KIAA1755*. Genetic risk scores were not associated with CV events in 357,882 unrelated individuals from UK Biobank. We also did not observe associations of QT dynamics during exercise and recovery with cardiovascular events. Increased QT dynamics during recovery was significantly associated with all-cause mortality in the univariate Cox regression analysis (hazard ratio [HR]: 1.09, 95% confidence interval [CI]: 1.05-1.13, P=2.28 x 10-5), but the association was not significant after adjusting for clinical risk factors.

Conclusions - QT interval dynamics during exercise and recovery are heritable markers but do not carry independent prognostic information for clinical outcomes in the UK Biobank, a population-based cohort. Their prognostic importance may relate to cardiovascular disease cohorts where structural heart disease and/or ischaemia may influence repolarization dynamics. The strong overlap between QT dynamics and resting QT interval loci suggests common biological pathways, however non-overlapping loci suggests alternative mechanisms may exists that underlie QT interval dynamics.

Circulation: Genomic and Precision Medicine

Key words: exercise; repolarization; Genome Wide Association Study; QT interval electrocardiography; cardiovascular disease risk factors; QT dynamics

Nonstandard Abbreviations and Acronyms:

ACM	All-cause mortality
CV	Cardiovascular
DBP	diastolic blood pressure
eQTL	expression quantitative trait locus
GRS	Genetic risk score
GTEx	Genotype-Tissue Expression
GWAS	Genome-wide association study
HES	Hospital episode statistics
LD	linkage disequilibrium
QC	Quality control
SBP	Systolic blood pressure
SNV	Single nucleotide variation
UCSC	University of California, Santa Cruz
UKB	UK Biobank
VEP	Variant effect predictor

American Heart Association.
Circulation: Genomic and Precision Medicine

Introduction

The electrocardiographic (ECG) QT interval reflects the total duration of ventricular depolarization and repolarization and is a biomarker for cardiovascular (CV) risk and death, with an estimated heritability of ~50% in twin-studies[1]. Characteristically, the interval shortens and prolongs with increasing and decreasing heart rate respectively. Multiple lines of evidence

indicate that abnormal dynamics of the QT interval, measured by the slope of the QT/RR profile, carries prognostic information in cardiac patients for CV mortality[2-9]. The genetic architecture of QT dynamics has not been investigated and might inform biological mechanisms that underlie QT dynamics. Furthermore, the prognostic value of QT dynamics in a population based cohort has not been evaluated.

An exercise stress test is a useful method to examine the dynamics of the QT interval in response to changes in heart rate. We first measured QT dynamics (approximated using RT dynamics) during exercise and recovery in 56,643 unselected individuals without a history of CV disease from the UK Biobank (UKB) study. We next conducted a genome-wide association studies (GWASs) in a subgroup of ~52,000 individuals of European ancestry. We report for the first time the genetic architecture of QT dynamics, genetic risk score results with CV outcomes and evaluation of QT dynamics with CV events and all-cause mortality.

Circulation: Genomic and Precision Medicine

Methods

The experimental design of the study is shown in Figure 1. Methods describing the derivation of QT dynamics, the genetic analysis, and survival analysis are available in the Supplemental Material. The UKB study has approval from the North West Multi-Centre Research Ethics Committee, and all participants provided informed consent[10]. Data used in this study were part of UKB application number 8256 and anonymized data and materials generated in this work have been returned to UKB and can be accessed per request.

Results

QT dynamics during exercise and recovery are heritable markers

An overview of the study results is provided in Figure 2. The demographics of the discovery and replication samples did not significantly differ (Supplementary Table 1). In the discovery phase, genome-wide association results of ~9.8 million SNVs from ~30,000 individuals of European ancestry from UKB were analyzed for each trait, QT dynamics during exercise and during recovery. All SNVs with $P < 1 \times 10^{-6}$ were compiled and these were organized into regions of 1Mb. The SNV with the lowest P value in each 1Mb region was selected as the lead SNV. In total, 20 lead SNVs for QT dynamics during exercise and 7 for QT dynamics during recovery were taken forward into replication in ~22,000 unrelated individuals. Twelve SNVs for QT dynamics during exercise formally replicated ($P \leq 0.05/20 = 0.0025$) and all had concordant directions of effect (Table 1). For QT dynamics during recovery, 2 SNVs were formally replicated ($P \leq 0.05/7 = 0.0071$), all with concordant directions of effect (Table 2). We next performed a full data set GWAS for each trait (Methods). Six additional SNVs reached genome-wide significance for QT dynamics during exercise and 1 SNV for QT dynamics during recovery, all with concordant directions of effect in the full GWAS data set (Supplemental Fig. 4, Table 1). The QQ plots for both markers (Supplemental Fig. 5) did not show evidence of population stratification or inflation. Regional plots are provided in Supplemental Fig. 6. Heritability estimations in the full dataset for QT dynamics during exercise and recovery were 10.7% and 5.4%, respectively, and their respective genetic correlation was 60%.

Conditional analysis revealed one secondary independent signal for QT dynamics during exercise at the *SCN5A-SCN10A* locus (rs6795970, Supplemental Fig. 7A). This signal was ~100 Kb downstream of the lead signal at this locus (rs7638275). For QT dynamics during recovery, one secondary signal was identified at the *NOS1AP* locus (rs16847548), at approximately 163 Kb upstream of the lead signal (rs12737539) (Supplemental Fig. 7B). Collectively, the lead and

American Heart Association.
Circulation: Genomic and Precision Medicine

secondary variants explained 2.1% of the variance of QT dynamics during exercise (~20% of heritability) and 0.6% (~11% of heritability) for QT dynamics during recovery.

Sex-stratified analyses revealed one additional genome-wide significant locus (*FOXN3*) for QT dynamics during exercise (Supplemental Table 3, Supplemental Fig. 6B): this locus at chromosome 14 was only genome-wide significant ($P \leq 5 \times 10^{-8}$) in males (rs796647867, $P = 2.9 \times 10^{-8}$). No sex specific loci for QT dynamics during recovery were identified. Altogether, 19 novel loci were associated with QT dynamics during exercise and 3 with QT dynamics during recovery. Two loci (*PRKC*1 and *KCNE1*) were common to both, thus we identified 20 novel unique loci for QT dynamics (Figure 2).

Genetic overlap between QT dynamics and other ECG markers

American Heart Association.

Fifteen of the 20 loci discovered for QT dynamics overlapped with loci previously reported for resting QT interval from published GWAS (Fig. 3 and Supplemental Table 4). Two loci had reported genome-wide phenotype-genotype associations with another ECG marker: the *CDKN1A* locus with QRS and JT duration and the *KIAA1755* locus with resting heart rate (Supplemental Table 5).

Circulation: Genomic and Precision Medicine

Functional annotation of QT dynamics loci and candidate genes

The lead variants or close proxies at the five unique loci for QT dynamics during exercise (*KCNQ4*, *CDKN1A*, *LOC643623*, *KIAA1755*, *FOXN3*) were all annotated as either intronic or intergenic (Supplemental Table 6), and no variants were associated with changes in expression levels of nearby genes in the GTEx database[11]. We explored the regulatory potential of the associated variants at these 5 loci using Hi-C datasets and observed significant chromatin interaction (RegulomeDB score = 2b) at *KIAA1755* with several genes in heart ventricles, brain, and adrenal gland (Supplemental Table 7).

At the remaining 15 loci for QT dynamics 5 lead variants or their close proxies mapping to *SLC4A3*, *SCN5A-SCN10A*, *KCNH2*, *FADS2, and KCNE1* were annotated as missense variants (Supplemental Table 6). The variant rs751489327, a close proxy of the lead variant at *CNOT1,* was annotated as a frameshift variant (Supplemental Table 6). The associated variants at *KCNH2* and *KCNE1* were predicted to be possibly damaging or deleterious by either SIFT or POLYPHEN[12]. Using COLOC[13], we observed evidence for colocalization (posterior probability > 75%) between significant gene expression (eQTL) and QT dynamics GWAS signals at two loci: *LITAF* and *PRKCA* in left ventricular and atrial appendage tissue respectively (Supplemental Table 8). Interestingly, variants at the *PRKCA* locus were discovered for both QT dynamics traits, and both demonstrated high degree of colocalization with the eQTL signal (Supplemental Fig. 8). The top eQTL variant for this locus in the heart left ventricle was rs11658550, this variant was in high LD (r2 > 0.9) with the lead variants for QT dynamics during exercise (rs569258685) and recovery (rs12960410).

American Heart Association.
Circulation: Genomic and Precision Medicine

DEPICT analysis indicated significant enrichment in heart and ventricular tissue (Supplemental Table 9A). Several genes including *SCN5A-SCN10A*, *SLC35F1*, and *KCNQ1* were indicated as prioritized genes (Supplemental Table 9B). Supplemental Table 10 presents an overview of potential candidate genes at each locus from all bioinformatic analyses and literature review. For these candidate genes, we found significant enrichments across several gene ontology terms including voltage gated channel activity and cardiac muscle cell action potential. Ventricular fibrillation and torsade de pointes were indicated as the human phenotypes. With fewer genes for QT dynamics during recovery, the enrichments included several ventricular membrane repolarization processes (Supplemental Table 11).

Genetic risk score analyses

Given the limited number of variants associated with QT dynamics during recovery, we only tested the GRS for QT dynamics during exercise for CV outcome. The GRS was constructed using 18 lead and 1 secondary SNVs (Table 1) discovered in the full GWAS analysis. From the 357,822 included individuals, 18,732 (5.2%) had a CV event. The CV risk in the top 5% was not higher compared to the bottom 5% of the GRS distribution: odds ratio: 1.05, CI = 0.96-1.16, P = 0.3.

Survival analyses

We also assessed whether QT dynamics is associated with CV outcome and ACM. Among the 55,643 individuals without a previous history of CV events in the exercise UKB cohort (Fig. 1), 1,786 (3.2%) had a CV event, and 999 (1.8%) died (Table 3). QT dynamics during exercise and recovery were not significantly different for the CV event group, but were significantly higher in the ACM group (median [interquartile range - IQR] values of 0.18 (0.07) vs. 0.17 (0.07), P = 0.04 for QT dynamics during exercise, and 0.12 (0.10) vs. 0.11 (0.09), P = 0.03, for QT dynamics during recovery). Only QT dynamics during recovery was a significant predictor of ACM in the univariate Cox regression analysis: hazard ratio (HR): 1.09 (confidence interval: 1.05 – 1.13), p = 2.28 x 10^{-5}, but this did not remain significant after adjusting for covariates (Table 4).

American Heart Association.
Circulation: Genomic and Precision Medicine

Discussion

This is the first study to systematically investigate the genetic basis and prognostic value of QT dynamics during exercise and recovery in a large cohort of unselected individuals without a history of CV events. Our main findings are: (1) QT dynamics during exercise has a significant heritable component (10%), with the recovery marker being less heritable (~6%); (2) there is

substantial genetic overlap between both QT dynamics markers and resting QT interval, and support for 5 loci for QT dynamics during exercise not previously reported for resting QT interval, and (3) QT dynamics during exercise and recovery were not associated with the occurrence of CV events or ACM in a population-based cohort.

Genetic architecture of exercise QT dynamics

We demonstrate for the first time that QT dynamics during exercise and recovery are heritable, however compared to the resting QT interval, the heritability is less and environmental factors likely have a more significant. Substantial overlap between loci for QT dynamics and resting QT interval suggests important genetic overlap. We observed variants at loci containing genes previously established to cause monogenic long QT syndrome and encoding ion channels or channel-interacting proteins (*KCNQ1*, *KCNH2*, *SCN5A-SCN10A*, and *KCNE1)*. This may suggest that common variants in these genes do not only play a role in modulating resting QT interval, but also QT dynamics, possibly by affecting rate-dependent channel kinetics. For example, *KCNQ1* and *KCNE1* are well known to confer kinetic properties on IKs currents required for rate adaptation of the cardiac action potential during exercise when β-adrenergic stimulation enhances IKs currents[14].

American Heart Association.
Circulation: Genomic and Precision Medicine

Five loci did not overlap with previously reported resting QT interval loci and thus may indicate biological mechanisms that specifically underlie QT dynamics. For example, one interesting candidate gene is *KCNQ4,* which encodes the potassium voltage-gated channel subfamily Q Member 4. *KCNQ4* is expressed in neurons and blood vessels and may also be involved in cardiac mitochondrial calcium handling[15]. The neuronal function is best established in the cochlear, where mutations are associated with human dominant hereditary deafness[16], but, this gene is widely expressed in other nerves and may regulate transmitter release in autonomic

ganglia and nerve terminals. A second potential candidate gene at a different locus is *KIAA1755*, which is only characterized at the transcriptional level[11]. Variants at this locus have previously been associated with resting heart rate[17] and heart rate variability[18]. *KIAA1755* is highly expressed in brain and nerve tissues and may play a role in the autonomic control of the heart.

From the loci that overlapped with resting QT interval, we found evidence for colocalization at the *PRKCA* locus between eQTL signals and GWAS signals for QT dynamics during exercise and recovery. This gene is an important regulator of cardiac contractility and *Ca*(2+) handling in myocytes. Mechanistically, modulation of PKC-alpha activity affects dephosphorylation of the sarcoplasmic reticulum Ca(2+) ATPase-2 (SERCA-2) pump inhibitory protein phospholamban (PLB), and alters sarcoplasmic reticulum Ca(2+) loading and the Ca(2+) transient. The regulation of cardiac contraction is critically important during exercise and recovery and is tightly coupled with the electrical processes of the heart, which may be reflected in the QT dynamics possibly through recognized Ca(2+) dependent mechanisms and stretch influencing QT interval[19].

American Heart Association.
Circulation: Genomic and Precision Medicine

Previous work has shown that ventricular repolarization is strongly modulated by heart rate, but also independently by autonomic nervous activity[20] and QT dynamics is thought to offer a more integrative measure of autonomic balance under stress than resting QT interval alone[21]. This is supported by multiple lines of evidence showing that abnormal QT dynamics is an independent predictor of CV mortality in cardiac patients[2-9].

The QT dynamics and the response of autonomic nervous activity have shown to be different between females and males[22]. The variant rs796647867 at the *FOXN3* locus was only associated with QT dynamics during exercise in men, suggesting that the genetic basis for QT dynamics during exercise may differ between sexes. *FOXN3* is a candidate gene at the locus and

is a member of the forkhead/winged helix transcription factor family. The mechanism by which it could modulate QT dynamics is unclear.

Prognostic value of QT dynamics in the general population

The independent association between QT dynamics and CV events has been established in multiple studies including heart failure[8], cardiomyopathy[9], and post myocardial infarction patients[2] but not in a population based sample as interrogated in our study. We found that QT dynamics was not an independent predictor of CV events and ACM. A review of the previous studies indicates only one used exercise stress data to measure QT dynamics during recovery[4]. According to this study, QT dynamics was a predictor of mortality in N=2,994 CV patients. Reported values for QT dynamics were higher compared to our findings (0.39 versus 0.17 and 0.11, respectively). Increased QT dynamics are observed in patients at risk for cardiac death and arrhythmic events[23] and the fact that our values were lower may explain why QT dynamics was not associated with CV outcome or mortality in our cohort. It is possible that we measured lower values for QT dynamics because our population was healthier. However, it should be mentioned that they exercised at submaximal level only. It is well known that maximal workloads are superior in predicting CV risk and ACM[24]. In addition, in our data the duration of the recovery period was shorter. The time-lag in the response of the QT interval to a sudden change in heart rate can take up to 2 minutes[25]. The recovery period in our work lasted 1 minute, and it is therefore possible that the QT interval was not fully adapted resulting in a reduced QT/RR slope during recovery. QT interval dynamics reported in this work are specific for exercise and recovery. Importantly, QT dynamics has also shown to carry prognostic value when derived from the QT/RR relationship of 24-h ECG recordings using linear regression between all QT and RR values. We did not have access to this data, but reported values in patients (0.17-0.22 for

American Heart Association.
Circulation: Genomic and Precision Medicine

heart failure patients[8] and 0.20 for cardiomyopathy[9]) were similar to QT dynamics during exercise observed in our cohort (0.17).

Limitations

Several limitations in our study should be noted. First, from the 22 SNVs discovered, 14 formally replicated. The remaining identified SNVs we are reporting are from a GWAS of all samples and require formal replication in an independent data set. We also note our sample is relatively small compared to many other GWAS and this will limit power for discovery of loci. Second, the power to evaluate the predictive value of QT dynamics was limited as the number of events was low. This coupled with the small number of genetic variants (N=19) which only explained 2.1% of the variance of QT dynamics during exercise (~20% of heritability), also affects the power of the GRS.

American Heart Association.

In summary, we demonstrate for the first time that QT dynamics during exercise and recovery are heritable markers. Its genetic basis largely overlaps with resting QT interval, however there may be additional biological mechanisms that specifically underlie QT dynamics having identified 5 novel loci specific to these traits . QT dynamics during exercise and recovery were not independent predictors of CV events or ACM as opposed to CV disease cohorts where structural heart disease and/or myocardial ischaemia affect repolarization dynamics. Future studies will be required to evaluate the prognostic value of the GRS in CV cohorts.

Circulation: Genomic and Precision Medicine

Sources of Funding: This research has been conducted using the UKB Resource (application 8256) and is supported by grant MR/N025083/1, by the National Institutes of Health Research (NIHR) Cardiovascular Biomedical Centre at Barts and The London, Queen Mary University of London (QMUL), by the People Programme of the European Union's Seventh Framework Programme grant n° 608765 and Marie Sklodowska-Curie grant n° 786833, by the University College London Hospital Biomedicine NIHR, Barts Heart Centre Biomedical Research Centre.

Dr. Young is funded by the Medical Research Council (Grant code MR/R017468/1). This research utilized Queen Mary's Apocrita High performance cluster facility, supported by QMUL Research-IT. http://doi.org/10.5281/zenodo.438045.

Disclosures: None.

References:

1. Carter N, Snieder H, Jeffery S, Saumarez R, Varma C, Antoniades L, Spector T. Qt interval in twins. *J Hum Hypertens.* 2000;14:389-390.

2. Jensen BT, Abildstrom SZ, Larroude CE, Agner E, Torp-Pedersen C, Nyvad O, Ottesen M, Wachtell K, Kanters JK. Qt dynamics in risk stratification after myocardial infarction. *Heart Rhythm*. 2005;2:357-364.

3. Pathak A, Curnier D, Fourcade J, Roncalli J, Stein PK, Hermant P, Bousquet M, Massabuau P, Sénard JM, Montastruc JL. Qt dynamicity: A prognostic factor for sudden cardiac death in chronic heart failure. *Eur J Heart Fail.* 2005;7:269-275.

4. Johnson NP, Holly TA, Goldberger JJ. Qt dynamics early after exercise as a predictor of mortality. *Heart Rhythm*. 2010;7:1077-1084.

5. Chevalier P, Burri H, Adeleine P, Kirkorian G, Lopez M, Leizorovicz A, André-Fouët X, Chapon P, Rubel P, Touboul P. Qt dynamicity and sudden death after myocardial infarction: Results of a long-term follow-up study. *J Cardiovasc Electrophysiol.* 2003;14:227-233.

6. Algra A, Tijssen J, Roelandt J, Pool J, Lubsen J. Qt interval variables from 24 hour electrocardiography and the two year risk of sudden death. *Heart*. 1993;70:43-48.

7. Extramiana F, Neyroud N, Huikuri HV, Koistinen MJ, Coumel P, Maison-Blanche P. Qt interval and arrhythmic risk assessment after myocardial infarction. *Am J Cardiol.* 1999;83:266-269, A266.

8. Cygankiewicz I, Zareba W, Vazquez R, Almendral J, Bayes-Genis A, Fiol M, Valdes M, Macaya C, Gonzale-Juanatey JR, Cinca J. Prognostic value of qt/rr slope in predicting mortality in patients with congestive heart failure. *J Cardiovasc Electrophysiol.* 2008;19:1066-1072.

9. Iacoviello M, Forleo C, Guida P, Romito R, Sorgente A, Sorrentino S, Catucci S, Mastropasqua F, Pitzalis M. Ventricular repolarization dynamicity provides independent prognostic information toward major arrhythmic events in patients with idiopathic dilated cardiomyopathy. *J Am Coll Cardiol.* 2007;50:225-231.

American Heart Association.
Circulation: Genomic and Precision Medicine

10. Sudlow C, Gallacher J, Allen N, Beral V, Burton P, Danesh J, Downey P, Elliott P, Green J, Landray M, et al. Uk biobank: An open access resource for identifying the causes of a wide range of complex diseases of middle and old age. *PLOS Medicine*. 2015;12:e1001779.

11. Consortium G. Genetic effects on gene expression across human tissues. *Nature*. 2017;550:204.

12. McLaren W, Gil L, Hunt SE, Riat HS, Ritchie GR, Thormann A, Flicek P, Cunningham F. The ensembl variant effect predictor. *Genome Biol*. 2016;17:122.

13. Giambartolomei C, Vukcevic D, Schadt EE, Franke L, Hingorani AD, Wallace C, Plagnol V. Bayesian test for colocalisation between pairs of genetic association studies using summary statistics. *PLoS Genet.* 2014;10:e1004383.

14. Harmer SC, Tinker A. The impact of recent advances in genetics in understanding disease mechanisms underlying the long qt syndromes. *Biol Chem.* 2016;397:679-693.

15. Testai L, Barrese V, Soldovieri MV, Ambrosino P, Martelli A, Vinciguerra I, Miceli F, Greenwood IA, Curtis MJ, Breschi MC. Expression and function of kv7. 4 channels in rat cardiac mitochondria: Possible targets for cardioprotection. *Cardiovasc Res.*2015;110:40-50.

16. Kubisch C, Schroeder BC, Friedrich T, Lütjohann B, El-Amraoui A, Marlin S, Petit C, Jentsch TJ. Kcnq4, a novel potassium channel expressed in sensory outer hair cells, is mutated in dominant deafness. *Cell*. 1999;96:437-446.

17. Den Hoed M, Eijgelsheim M, Esko T, Brundel BJ, Peal DS, Evans DM, Nolte IM, Segrè AV, Holm H, Handsaker RE. Identification of heart rate–associated loci and their effects on cardiac conduction and rhythm disorders. *Nat Genet.* 2013;45:621.

18. Nolte IM, Munoz ML, Tragante V, Amare AT, Jansen R, Vaez A, von der Heyde B, Avery CL, Bis JC, DierchxB, et al. Genetic loci associated with heart rate variability and their effects on cardiac disease risk. Nat Commun. 2017;8:15805.

19. Qu Z, Nivala M, Weiss JN. Calcium alternans in cardiac myocytes: Order from disorder. *J Mol Cell Cardiol.* 2013;58:100-109.

20. Susmano A. Effect of heart rate and autonomic tone on the qt interval. *Circulation*. 1982;66:478-478.

21. Sundaram S, Carnethon M, Polito K, Kadish AH, Goldberger JJ. Autonomic effects on qt-rr interval dynamics after exercise. *Am J Physiol Heart Circ Physiol.* 2008;294:H490-H497.

22. Dart AM, Du X-J, Kingwell BA. Gender, sex hormones and autonomic nervous control of the cardiovascular system. *Cardiovasc Res.* 2002;53:678-687.

Circulation: Genomic and Precision Medicine
American Heart Association.

23. Zareba W, De Luna AB. Qt dynamics and variability. *Ann Noninvasive Electrocardiol*. 2005;10:256-262.

24. Qiu S, Cai X, Sun Z, Li L, Zuegel M, Steinacker JM, Schumann U. Heart rate recovery and risk of cardiovascular events and all-cause mortality: A meta-analysis of prospective cohort studies. *J Am Heart Assoc*. 2017;6:e005505.

25. Franz MR, Swerdlow CD, Liem LB, Schaefer J. Cycle length dependence of human action potential duration in vivo. Effects of single extrastimuli, sudden sustained rate acceleration and deceleration, and different steady-state frequencies. *J Clin Invest.* 1988;82:972-979.

American Heart Association.

Circulation: Genomic and Precision Medicine

Table 1. Loci associated with QT dynamics during exercise

						Discovery				Replication				Full			
**Locus**	**SNV**	**CHR**	**BP**	**EA**	**EAF**	**P**	**n**	**beta**	**SE**	**P**	**n**	**beta**	**SE**	**P**	**n**	**beta**	**SE**
**RPL22**	**rs10864434**	**1**	**6262231**	**A**	**0.602**	**6.40E-18**	**28346**	**-0.062**	**0.007**	**1.40E-11**	**21086**	**-0.059**	**0.005**	**2.90E-27**	**49935**	**-0.059**	**0.005**
KCNQ4	rs116015634	1	41250961	C	0.977	6.70E-06	29453	0.103	0.023	7.20E-04	21909	0.097	0.017	2.30E-08	51884	0.097	0.017
**NME7**	**rs1591734**	**1**	**169271902**	**C**	**0.860**	**2.50E-13**	**29428**	**0.073**	**0.010**	**1.10E-08**	**21890**	**0.069**	**0.008**	**4.30E-20**	**51839**	**0.069**	**0.008**
SLC4A3	rs35394392	2	220500830	G	0.780	1.90E-06	29335	-0.040	0.008	4.70E-04	21821	-0.036	0.006	7.50E-09	51676	-0.036	0.006
**SCN5A-SCN10A†**	**rs7638275**	**3**	**38665823**	**A**	**0.019**	**5.50E-14**	**28415**	**0.193**	**0.026**	**9.80E-05**	**21137**	**0.165**	**0.019**	**2.40E-17**	**50055**	**0.165**	**0.019**
**CDKN1A**	**rs1321311**	**6**	**36622900**	**C**	**0.764**	**1.10E-09**	**29453**	**0.050**	**0.008**	**3.00E-05**	**21909**	**0.045**	**0.006**	**1.60E-13**	**51884**	**0.045**	**0.006**
**SLC35F1**	**rs28436726**	**6**	**118664854**	**G**	**0.940**	**9.20E-18**	**29186**	**0.126**	**0.015**	**6.60E-06**	**21710**	**0.107**	**0.011**	**3.70E-22**	**51414**	**0.107**	**0.011**
**LOC643623**	**rs35846768**	**6**	**126024943**	**C**	**0.707**	**8.10E-08**	**26148**	**-0.043**	**0.008**	**9.90E-05**	**19451**	**-0.041**	**0.006**	**2.50E-11**	**46063**	**-0.041**	**0.006**
KCNH2	rs2072412	7	150647970	C	0.729	9.10E-06	29034	0.035	0.008	4.40E-05	21598	0.035	0.006	1.70E-09	51146	0.035	0.006
KCNQ1	rs2074238	11	2484803	T	0.088	1.30E-05	29453	0.052	0.012	1.30E-08	21909	0.066	0.009	6.10E-13	51884	0.066	0.009
FADS2	rs174594	11	61619829	C	0.376	1.30E-05	29283	-0.031	0.007	1.50E-03	21783	-0.029	0.005	4.60E-08	51585	-0.029	0.005
**KLF12**	**13:74505951_CT_C**	**13**	**74518210**	**G**	**0.631**	**4.40E-10**	**29418**	**-0.045**	**0.007**	**3.30E-06**	**21883**	**-0.039**	**0.008**	**2.70E-14**	**51823**	**-0.041**	**0.005**
FOXN3§	rs796647867	14	90168955	ATT	0.592	-	-	-	-	-	-	-	-	2.90E-08	23724	-0.047	0.008
**LITAF**	**rs8049607**	**16**	**11691753**	**T**	**0.515**	**1.90E-09**	**29453**	**0.042**	**0.007**	**3.80E-05**	**21909**	**0.039**	**0.005**	**8.20E-14**	**51884**	**0.039**	**0.005**
**CNOT1**	**16:58565409_TGA_T**	**16**	**58566671**	**A**	**0.752**	**7.20E-10**	**29453**	**0.050**	**0.008**	**2.60E-14**	**21909**	**0.071**	**0.009**	**2.60E-23**	**51884**	**0.060**	**0.006**
**PRKCA***	**rs569258685**	**17**	**64313077**	**G**	**0.421**	**3.80E-06**	**29360**	**-0.032**	**0.007**	**4.40E-07**	**21840**	**-0.042**	**0.008**	**9.30E-12**	**51719**	**-0.036**	**0.005**
**KCNJ2**	**rs12941598**	**17**	**68453327**	**G**	**0.786**	**1.00E-08**	**29287**	**0.048**	**0.008**	**2.30E-05**	**21785**	**0.046**	**0.006**	**4.90E-13**	**51591**	**0.046**	**0.006**
KIAA1755	rs6069151	20	36830133	A	0.481	4.40E-06	28982	-0.032	0.007	3.90E-03	21558	-0.029	0.005	3.30E-08	51054	-0.029	0.005
**KCNE1**	**rs1805128**	**21**	**35821680**	**C**	**0.986**	**3.10E-09**	**29453**	**-0.177**	**0.030**	**4.50E-08**	**21909**	**-0.182**	**0.022**	**4.50E-16**	**51884**	**-0.182**	**0.022**

The locus name indicates the gene that is in the closest proximity to the most associated SNV. Replicated SNVs are indicated in bold type
* indicates the SNV is the same or in high LD (r2 > 0.8) with a SNV associated with the QT dynamics during exercise or recovery trait
SNV single-nucleotide polymorphism, CHR Chromosome, BP Base pair Position, based on HG build 19, EA Effect allele, EAF Effect allele frequency from discovery data set, β Beta in beats per minute, SE Standard Error, N effective number of participants, P P-value.
† Secondary SNV identified (rs6795970) at SCN5A-SCN10A locus using conditional analysis.
§ Sex-specific locus: Association was only significant in males.

Circulation: Genomic and Precision Medicine
American Heart Association.

Table 2. Loci associated with QT dynamics during recovery

						Discovery				Replication				Full			
Locus	SNV	CHR	BP	EA	EAF	P	n	beta	SE	P	n	beta	SE	P	n	beta	SE
**NOS1AP†**	**rs12737539**	**1**	**162198429**	**G**	**0.364**	**2.70E-07**	**28980**	**0.040**	**0.008**	**4.30E-03**	**21608**	**0.026**	**0.009**	**4.60E-09**	**51107**	**0.034**	**0.006**
**PRKCA***	**rs12940610**	**17**	**64312463**	**A**	**0.422**	**1.20E-09**	**29146**	**0.046**	**0.008**	**7.00E-04**	**21732**	**0.030**	**0.009**	**7.40E-12**	**51399**	**0.039**	**0.006**
KCNE1	rs41312993	21	35819445	A	0.967	1.40E-11	29205	-0.141	0.021	2.20E-01	21776	-0.030	0.024	8.40E-09	51503	-0.091	0.016

The locus name indicates the gene that is in the closest proximity to the most associated SNV. Replicated SNVs are indicated in bold type
* indicates the SNV is the same or in high LD (r2 > 0.8) with a SNV associated with the QT dynamics during exercise or recovery trait
SNV single-nucleotide polymorphism, CHR Chromosome, BP Base pair Position, based on HG build 19, EA Effect allele, EAF Effect allele frequency from discovery data set, β Beta in beats per minute, SE Standard Error, N effective number of participants, P P-value.
† Secondary SNV identified (rs16847548) at NOS1AP locus using conditional analysis.

American Heart Association.
Circulation: Genomic and Precision Medicine

Table 3. Baseline characteristics stratified by outcome.

	Cardiovascular event				All-cause mortality		
Clinical Variables	Event (N=1,786)	Event free (N=53,857)	P		Event (N=999)	Event free (N=54,644)	P
Age (yr)	62 (57 - 66)	58 (50 - 63)	3.14E-106		63 (58 - 66)	58 (50 - 63)	3.61E-75
Sex (% man)	1275 (71)	24638 (46)	3.49E-103		608 (61)	25305 (46)	7.70E-20
BMI	28 (25 - 31)	26 (24 - 29)	1.95E-44		27 (25 - 30)	26 (24 - 29)	1.75E-05
Current smoker or history of smoking	921 (52)	22957 (43)	9.13E-14		587 (59)	23291 (43)	4.73E-24
Diabetes	192 (11)	2189 (4)	7.78E-32		83 (8)	2298 (4)	1.29E-08
Systolic blood pressure (mmHg)	143 (132 - 154)	136 (124 - 148)	3.87E-60		142 (130 - 152)	136 (124 - 148)	6.19E-20
Diastolic blood pressure (mmHg)	84 (77 - 90)	81 (75 - 88)	3.09E-22		82 (76 - 88)	82 (75 - 88)	1.10E-04
ECG variables							
Resting heart rate (bpm)	72 (63 - 80)	70 (63 - 78)	2.91E-05		72 (65 - 81)	70 (63 - 78)	7.12E-09
Heart rate increase during exercise (bpm)	37 (29 - 44)	41 (33 - 49)	2.13E-41		38 (30 - 46)	41 (33 - 49)	1.26E-13
Heart rate decrease during recovery (bpm)	23 (18 - 30)	27 (21 - 34)	2.90E-63		23 (17 - 30)	27 (21 - 34)	4.74E-35
QT dynamics during exercise (ms)	0.17 (0.13 - 0.21)	0.17 (0.14 - 0.21)	8.01E-01		0.18 (0.14 - 0.21)	0.17 (0.14 - 0.21)	3.92E-02
QT dynamics during recovery (ms)	0.11 (0.07 - 0.17)	0.11 (0.07 - 0.16)	9.33E-01		0.12 (0.07 - 0.17)	0.11 (0.07 - 0.16)	3.33E-02
Corrected QT interval (ms)	400 (383 - 417)	396 (381 - 411)	3.26E-10		400 (385 - 416)	396 (381 - 412)	7.86E-08

Comparison of clinical and ECG characteristics stratified by cardiovascular event and all-cause mortality. The corrected QT interval was calculated with the Bazett formula. The statistical significance was tested using the Wilcoxon rank-sum test. P-value < 0.05 were considered significant and are highlighted by the bold font. Values are median (interquartile range) or n (%). BMI = body mass index.

Circulation: Genomic and Precision Medicine

Table 4. Univariate and multivariate association of QT dynamics and other risk markers with all-cause mortality

	Univariate		Multivariate	
	HR (95% CI)	p	HR (95% CI)	p
**Clinical Variables**				
Age [per 1 SD]	2.05 (1.9-2.22)	**7.05E-80**	1.89 (1.74-2.06)	**3.61E-47**
Sex (male)	1.8 (1.59-2.05)	**3.02E-20**	1.68 (1.47-1.93)	**8.17E-14**
BMI [per 1SD]	1.1 (1.04-1.17)	**1.65E-03**	0.99 (0.92-1.06)	7.10E-01
Smoking (yes)	1.9 (1.67-2.15)	**4.22E-24**	1.58 (1.39-1.8)	**2.37E-12**
Diabetes (yes)	2.05 (1.63-2.56)	**1.78E-10**	1.28 (1.02-1.62)	**3.62E-02**
SBP [per 1 SD]	1.32 (1.24-1.4)	**2.29E-19**	1.04 (0.95-1.14)	3.57E-01
DBP [per 1 SD]	1.12 (1.05-1.19)	**3.89E-04**	0.97 (0.89-1.06)	5.45E-01
**ECG variables**				
Resting HR [per 1 SD]	1.22 (1.15-1.29)	**7.79E-11**	1.16 (1.07-1.24)	**1.07E-04**
HR response to exercise [per 1 SD]	0.77 (0.72-0.82)	**1.37E-15**	1.21 (1.1-1.32)	**3.33E-05**
HR response to recovery [per 1 SD]	0.64 (0.6-0.69)	**8.26E-38**	0.79 (0.72-0.87)	**1.10E-06**
QT dynamics during exercise [per 1 SD]	1.02 (0.98-1.07)	3.62E-01		
QT dynamics during recovery [per 1 SD]	1.09 (1.05-1.13)	**2.28E-05**	1.04 (1-1.08)	6.26E-02
Corrected QT [per 1 SD]	1.19 (1.12-1.26)	**1.09E-08**	1.07 (1-1.15)	6.41E-02

Circulation: Genomic and Precision Medicine

Figure Legends:

Figure 1. Flowchart on selection of individuals for prognostic and genetic analyses of QT dynamics. QT dynamics were derived from the exercise cohort. Yellow panel presents the data selection for the prognostic analysis, blue presents the selection for the genome wide association studies (GWAS) and brown represents the individuals that were unrelated to individuals included in the GWAS to allow unbiased genetic risk analyses using the variants discovered in the GWAS. QC = quality control, CV = cardiovascular.

Figure 2. Association results of the QT dynamics GWAS in the full data. SNV: single-nucleotide variant. Note: To ensure there was no overlap between the discovery and the replication cohorts, we removed first and second-degree related individuals (kinship coefficient > 0.88).

American Heart Association.
Circulation: Genomic and Precision Medicine

Figure 3. Overlap between loci for QT dynamics during exercise (blue box) and recovery (yellow box) and reported loci for resting QT interval (black box). A substantial proportion of the loci for QT dynamics overlapped loci previously reported for resting QT interval. Five loci (bold) for QT dynamics during exercise did not overlap with previously reported loci for resting QT interval. All loci for QT dynamics during recovery overlapped with resting QT interval loci. *: sex-specific (males) locus for QT dynamics during exercise.

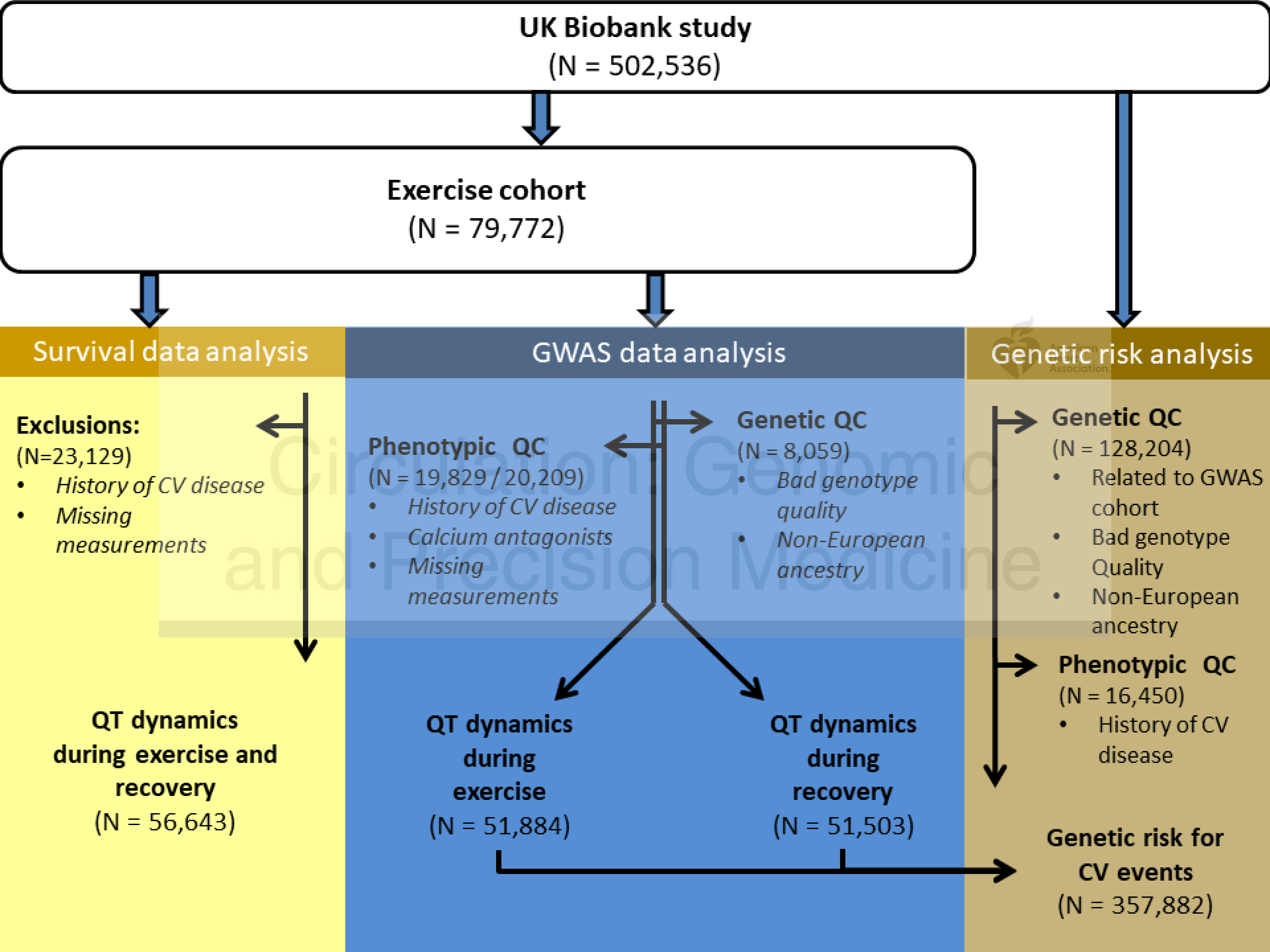

Circulation: Genomic and Precision Medicine

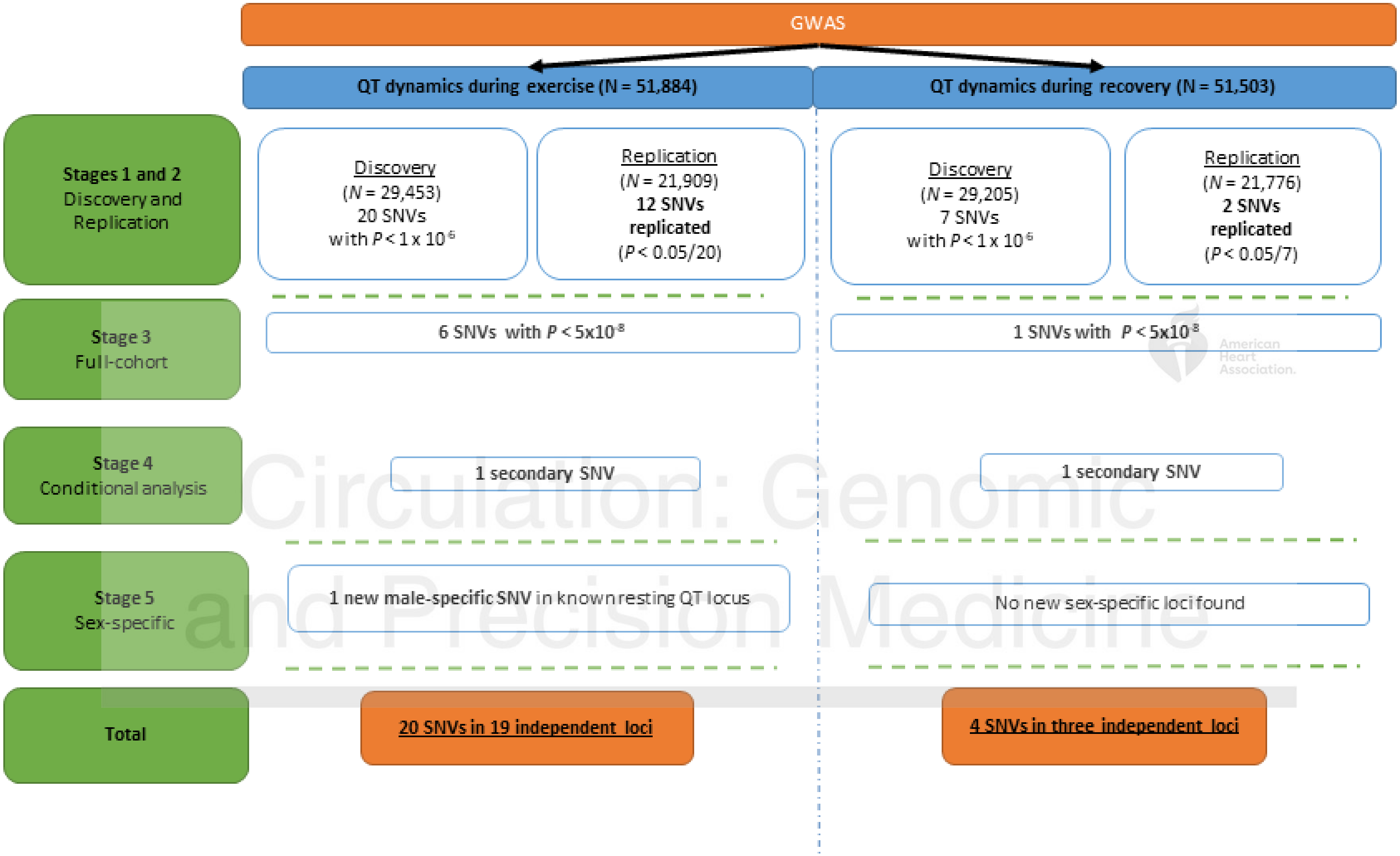

American Heart Association.
Circulation: Genomic and Precision Medicine

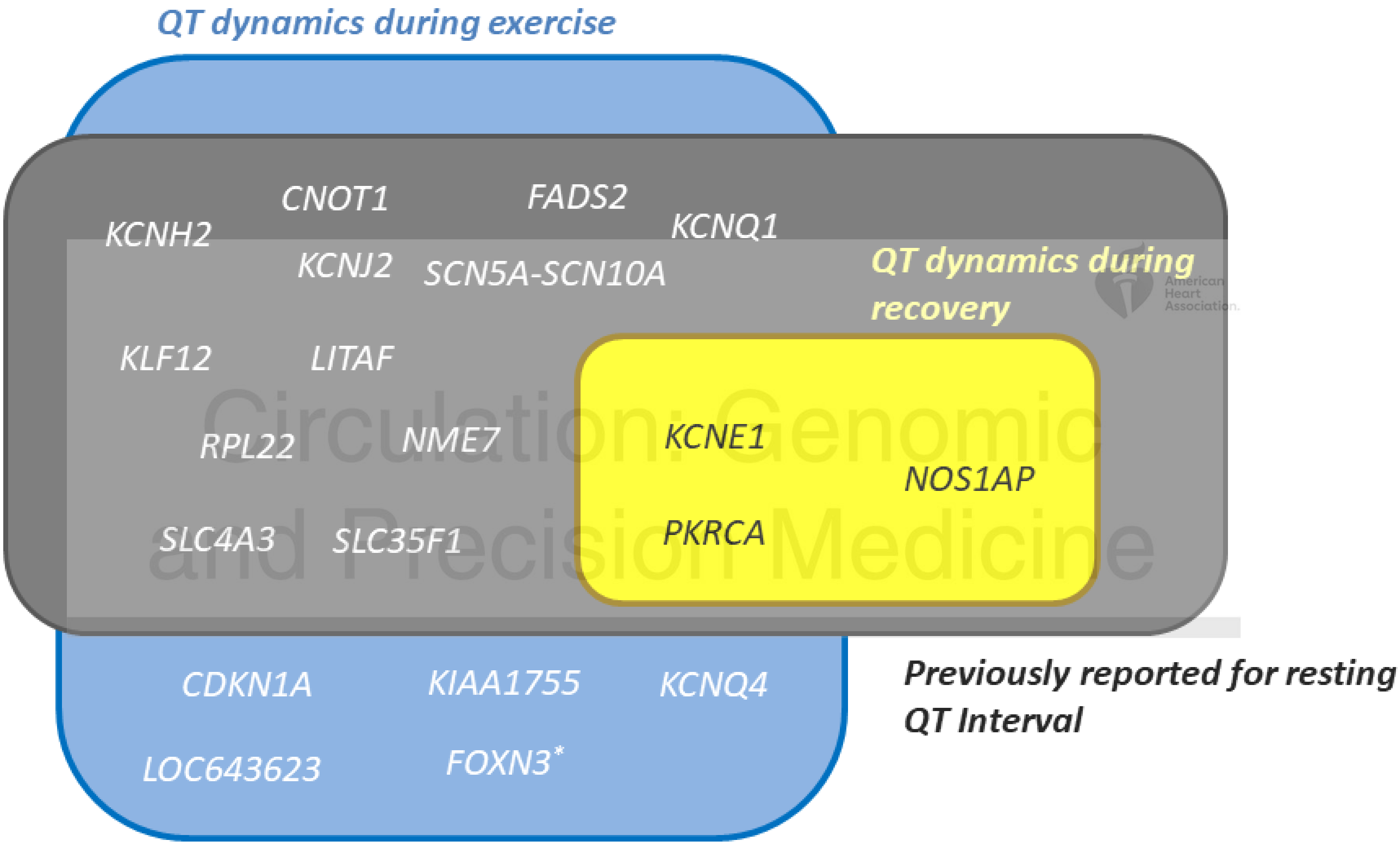

Circulation: Genomic and Precision Medicine
American Heart Association.
